# Neuropathic pain: Mechanisms and therapeutic strategies

**DOI:** 10.3389/fcell.2023.1072629

**Published:** 2023-01-16

**Authors:** Georg A. Petroianu, Lujain Aloum, Abdu Adem

**Affiliations:** Department of Pharmacology, College of Medicine and Health Science, Khalifa University of Science and Technology, Abu Dhabi, United Arab Emirates

**Keywords:** chronic pain, nociceptive pain, neuropathic pain, ATP, adenosine, capsaicin, clodronate, pregabalin

## Abstract

The physiopathology and neurotransmission of pain are of an owe inspiring complexity. Our ability to satisfactorily suppress neuropathic or other forms of chronic pain is limited. The number of pharmacodynamically distinct and clinically available medications is low and the successes achieved modest. Pain Medicine practitioners are confronted with the ethical dichotomy imposed by Hippocrates: On one hand the mandate of *primum non nocere*, on the other hand, the promise of heavenly joys if successful *divinum est opus sedare dolorem*. We briefly summarize the concepts associated with nociceptive pain from nociceptive input (afferents from periphery), modulatory output [descending noradrenergic (NE) and serotoninergic (5-HT) fibers] to local control. The local control is comprised of the “*inflammatory soup*” at the site of pain origin and synaptic relay stations, with an ATP-rich environment promoting inflammation and nociception while an adenosine-rich environment having the opposite effect. Subsequently, we address the transition from nociceptor pain to neuropathic pain (independent of nociceptor activation) and the process of sensitization and pain chronification (transient pain progressing into persistent pain). Having sketched a model of pain perception and processing we attempt to identify the sites and modes of action of clinically available drugs used in chronic pain treatment, focusing on adjuvant (co-analgesic) medication.

## 1 Introduction

The neurotransmission of pain is of an owe inspiring complexity. In contrast, our ability to satisfactorily suppress neuropathic and/or chronic pain is limited. The number of pharmacodynamically distinct available medications is low, and the therapeutical successes achieved modest. In the words of Carl Edward Noe **….** *when pain leads to suffering, it ceases to be a teacher and becomes the oldest medical malady* (Noe, 2020). Pain Medicine practitioners are confronted with the ethical dilemma imposed by Hippocrates, the delicate balance act between the mandate of *primum non nocere*, and the promise of heavenly joys if successful in delivering relief *divinum est opus sedare dolorem*.

Upon reviewing the current understanding of pain perception and processing we highlight the possible sites and modes of action of clinically available drugs used in pain treatment, focusing on adjuvant (co-analgesic) medication.

## 2 Pain: A simplified model

**FIGURE 1:**
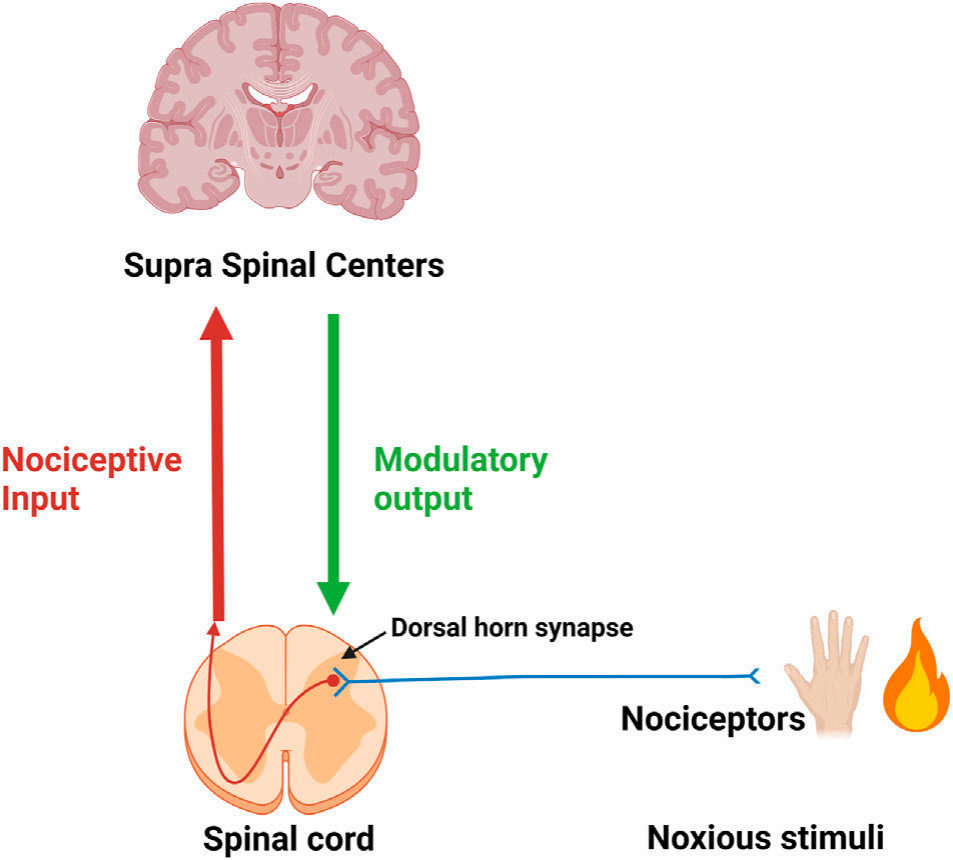
First neuron (in blue) of the ascending pain pathway. Red-brown arrow: Second ascending neuron leading to supra-spinal centers. Green arrow: Descending modulatory output from supra-spinal centers.

Noxious stimuli in the periphery are perceived *via* nociceptors and the information is relayed to the dorsal horn where the first synapse of the ascending pain pathway is localized. Post-synaptically the nociceptive input is relayed to higher (supra-spinal) centers. Modulating descending output from supra-spinal centers is received at dorsal horn level. The micro-environment (inflammatory soup) at the site of origin of noxious stimuli (injury) and around the dorsal horn have a significant influence on the intensity and duration of the signal (Figure 1).

### 2.1 Nociceptive input

Injury leads to activation of Transient Receptor Potential (TRP) nociceptors in the periphery; subsequent opening of cation channels results in depolarization and action potential propagation along afferent sensory fibers to the dorsal horn synapse. Presynaptic vesicles release excitatory non-peptide transmitter (glutamate, AMPA and NMDA receptor agonist), and peptide transmitters [Substance P (SP) (NK1 neurokinin receptor 1 agonist) and Calcitonin Gene-Related Peptide (CGRP) (Calcitonin like Receptor and Receptor Activity Modifying Protein complex agonist)]. Neuropeptide Y (NPY) and CGRP receptors co-localize extensively; the two neurotransmitters have mostly opposite effects (Nelson and Taylor, 2021). NPY acts at Y2 receptors on the central terminals of primary afferents to inhibit SP release (Duggan et al., 1991).

In blood vessels, CGRP acts as a potent vasodilator when compared to several known vasodilators such as histamine, prostaglandin E2 and SP. Inhibitors of the CGRP receptor are identified by the suffix–*gepant* (ubrogepant; atogepant). AMPA receptor antagonists are identified by the suffix*–ampanel* (perampanel) (De Caro et al., 2020). Kynurenic acid is one of the endogenous antagonists at ionotropic AMPA, NMDA (glycine-site ligand), and kainate glutamate receptors.

Second neuron: After crossing to the contralateral side, the signal travels in the ascending lateral spino-thalamic tract (TST) to the thalamus.

**FIGURE 2:**
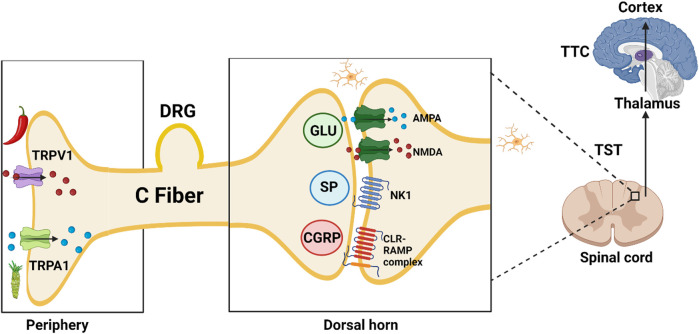
TRPV1, transient receptor potential vanilloid subfamily, member 1, capsaicin or hot chili pepper receptor; TRPA1, transient receptor potential ankyrin subfamily, member 1, allyl isothiocyanate or wasabi receptor; DRG, dorsal root ganglion; GLU, glutamate; SP, substance P (11 AA); AMPA, α-amino-3-hydroxy-5-methyl-4-isoxazolepropionic acid; NMDA, N-methyl-d-aspartate; NK1, Neurokinin 1 receptor; CGRP, calcitonin gene-related peptide (37 AA); CLR-RAMP, calcitonin like receptor—receptor activity modifying protein complex; TST, Tractus spino-thalamicus; TTC, tractus thalamo-corticalis; 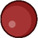
Ca^2+^; 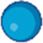
Na^+^; 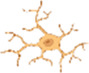
Microglia; 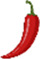
Chili pepper: Capsaicin; 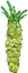
Wasabi: Allyl isothiocyanate.

Third neuron: From the thalamus to the sensory cortex in the parietal lobe in the thalamo-cortical tract TTC (Brodmann areas 1, 2, and 3) allowing pain localization (Figure 2).

### 2.2 Modulatory output

Descending noradrenergic (norepinephrine; NE) and serotoninergic (5-HT) fibers influence pain perception. Most noradrenergic fibers originate from the pontine locus coeruleus (LC; blue spot) while descending serotonergic pathways originate from the floor of the medulla oblongata from the nucleus raphe magnus (NRM). Both NE and 5-HT induce membrane hyperpolarization while decreasing the excitatory transmitter release from primary Aδ and C afferent fibers pre-synaptically and increasing the release of inhibitory GABA and glycine from interneurons (Yoshimura and Furue, 2006). The administration of 5-HT produces membrane hyperpolarization in about 50% of dorsal horn neurons, while NE hyperpolarizes more than 80% of them, suggesting the need to augment both NE and 5-HT concentrations in order to suppress algesia. Neither atomoxetine (selective noradrenaline reuptake inhibitor) nor an SSRI alone have such a marked effect (Miyahara et al., 2019).

Norepinephrine exerts *via* α2-adrenoceptors an inhibitory influence on neuropathic pain while α1-adrenoceptors exacerbate it (Kim et al., 2005).

**FIGURE 3:**
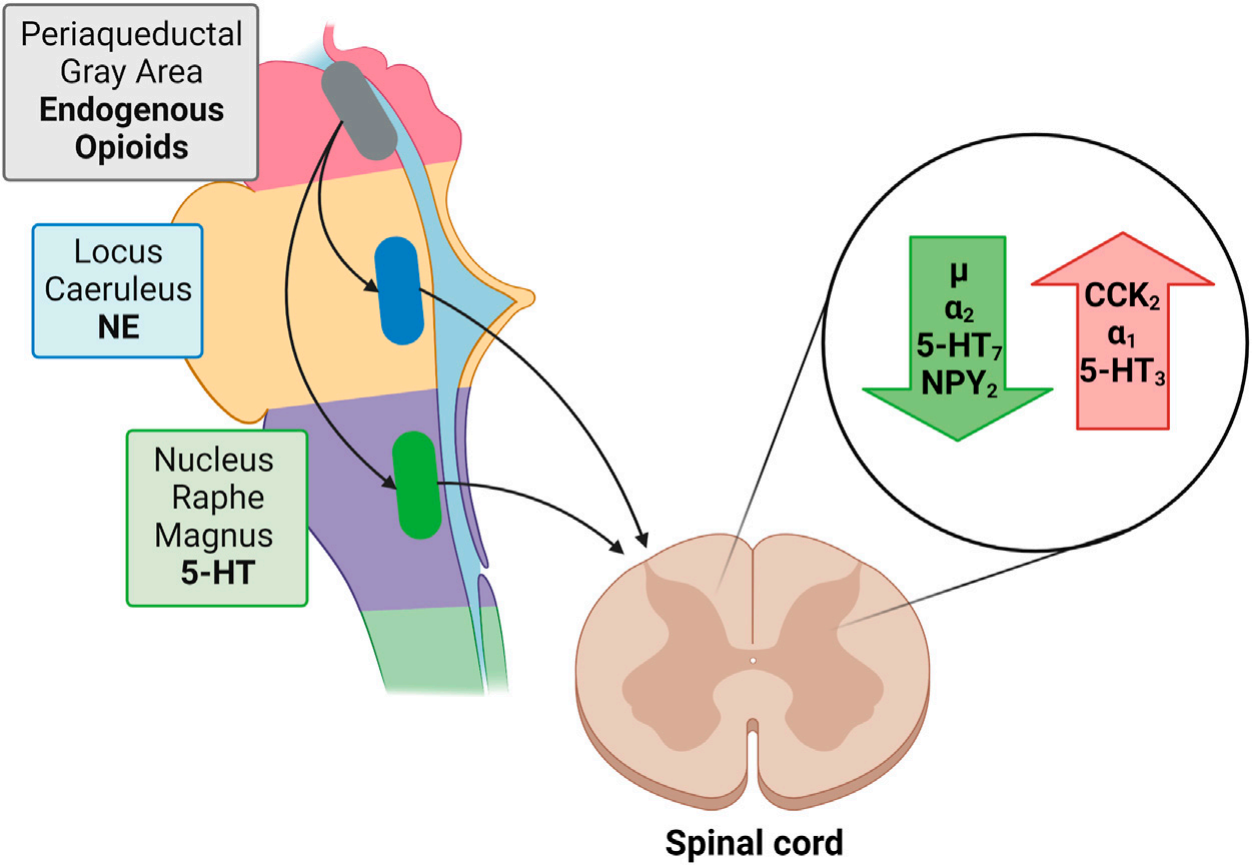
Green arrow: Receptors reducing nociception: µ opioid receptor; α_2_, adrenergic receptor; 5-HT_7_, serotonin receptor; NPY_2_, neuropeptide Y receptor type 2. Red arrow: Receptors enhancing nociception: CCK_2_, cholecystokinin 2(B) receptor; α_1_, adrenergic receptor; 5-HT_3_, ionotropic serotonin receptor; 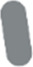
Periaqueductal Gray: Endogenous Opioids; 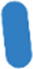
Locus Caeruleus: NE; 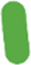
Nucleus Raphe Magnus: 5-HT.

As to the receptors involved in serotoninergic pain modulation findings indicate a role for 5-HT_7_ receptors in antinociception, and a role for 5-HT_3_ in pro-nociceptive facilitation (Dogrul et al., 2009). Activation of 5-HT_7_ receptors does not directly inhibit nociceptive dorsal horn neurons because these receptors are positively coupled to adenylate cyclase and their stimulation is excitatory. However, activation of 5-HT_7_ receptors localized on spinal inhibitory enkephalinergic or GABAergic interneurons, to evoke the release of enkephalins or GABA, produces an inhibition of nociceptive transmission (Brenchat et al., 2009; Liu et al., 2020) (Figure 3).

### 2.3 Local control

The activity of the feed-back loop consisting of (afferent) nociceptive input, and modulatory (efferent) output is markedly influenced by the composition of the “*cytokine soup*” at the site of origin and synaptic relay stations, with an ATP-rich environment promoting inflammation and nociception while an adenosine-rich environment having the opposite effect.

In an injured environment glia cells release intracellular ATP *via* the vesicular nucleotide transporter (VNUT) into the extracellular space. ATP activates purinergic P2X ionotropic and metabotropic P2Y receptors that increase the sodium and calcium conductance of various TRP receptors. Cell surface (ecto)nucleotidases by degrading nucleotides serve to terminate purinergic signaling; their function is analogous to the activity of cholinesterase at cholinergic synapses (Liu et al., 2017; Stokes et al., 2017).

**FIGURE 4:**
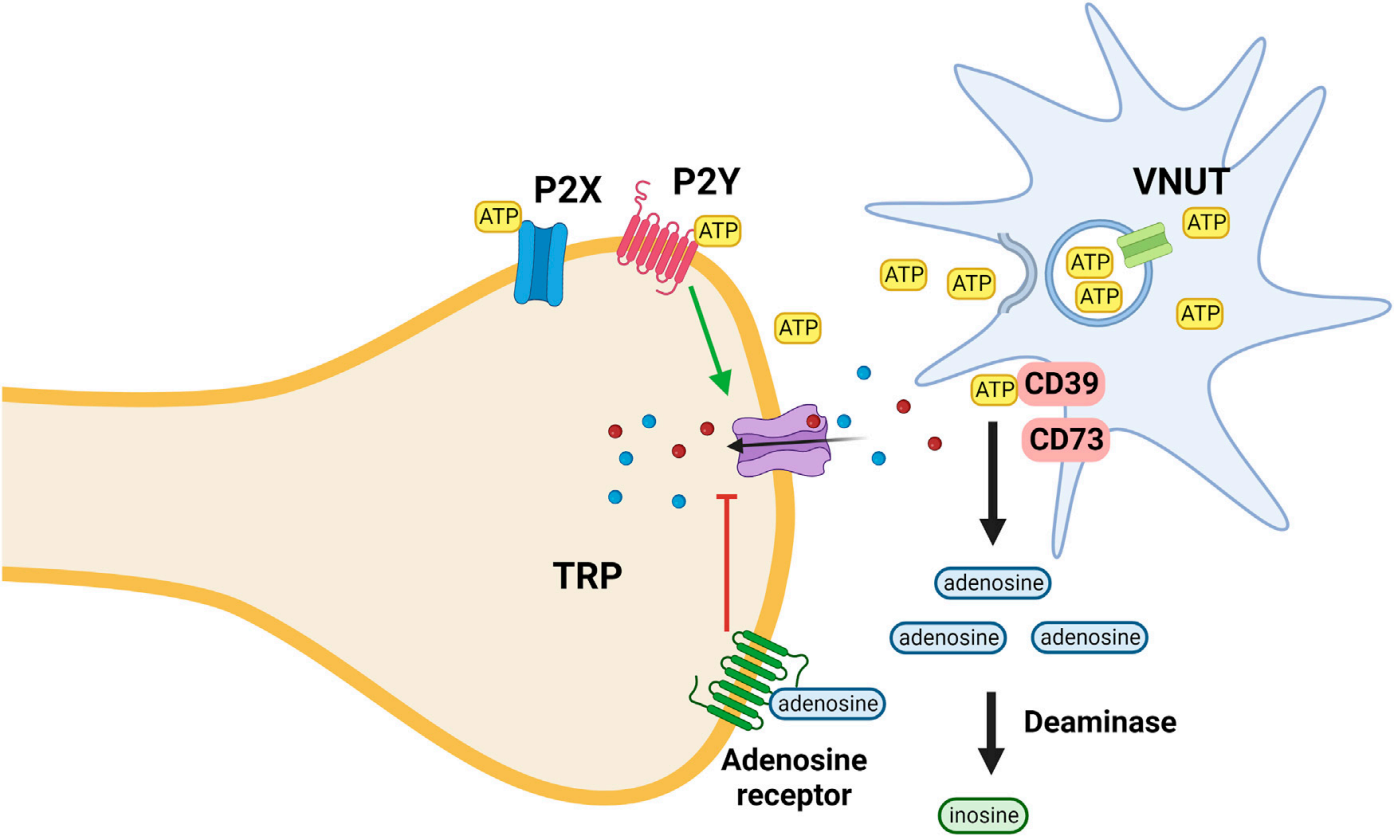
ATP is released from microglia, keratinocytes and other cells *via* the activity of the VNUT; inhibition of the transporter reduces the ATP concentration and thus its pro-inflammatory effect mediated *via* interaction with both ionotropic (P2X) and metabotropic (P2Y) purinergic receptors. ATP is metabolized by ectoenzymes (CD; cluster of differentiation 73 and 39). Extracellular adenosine generated by metabolic break-down of nucleotides activates adenosine receptors that decrease the sodium and calcium permeability of TRP receptors. Adenosine is metabolized by deaminases.

Extracellular adenosine generated by metabolic break-down of nucleotides activates adenosine receptors (A1, A2A, A2B, and A3) that decrease the sodium and calcium permeability of TRP receptors (Figure 4) (Battastini et al., 2021). The antinociceptive activity of adenosine is mediated mainly *via* activation of adenosine A1 receptors; activation of A3A receptors seems to play a role as well. However, the function of adenosine A2A and A2B receptors is more debatable as their activation causes both nociception and anti-nociception effects (Vincenzi et al., 2020).

The purinergic influence is complemented by a plethora of pro- and anti-nociceptive substances, both oligopeptide [endogenous opioid peptides, the anti-opioid cholecystokinin (CCK), bradykinin, and cytokines] and non-peptide (histamine, prostanoids, leukotrienes).

Non-steroidal anti-inflammatory drugs (NSAIDs) are effective in nociceptive pain and controlling pain associated with inflammation *via* inhibition of cyclooxygenase-2. They are commonly prescribed for neuropathic pain however; there is not sufficient evidence to support the usage of NSAIDs for such pain and thus are not listed in the major guidelines addressing the treatment of neuropathic pain (Vane and Botting, 1998; Cohen and Mao, 2014; Moore et al., 2015; Kappelmann et al., 2018). One has to consider however that pain is rarely exclusively neuropathic or nociceptive.

### 2.4 Neuropathic pain

Nociceptive pain is the physiologic response to nociceptor activation, and -as it is conducive to protective responses-it serves a useful purpose. Pain independent of nociceptor activation is called neuropathic; it is -most likely-not beneficial and it is the result of sensitization and autonomous ectopic activity of various pro-nociceptive entities, at the most basic level sodium and calcium channels. Dull nociceptive pain is contrasted by the sharp neuropathic one. There is overlap between chronic and neuropathic pain: neuropathic pain is chronic but not all chronic pain is neuropathic.

Most likely as a result of increased activity of the endogenous pro-nociceptive neuropeptide CCK, neuropathic pain does not respond well to opioids (Wiesenfeld-Hallin et al., 2002). The predominant form of CCK in the central nervous system is an octapeptide (Bowers et al., 2012). Opioid-induced CCK release is one of the postulated mechanisms explaining opioid tolerance and hyperalgesia (Smith, 2012). Recently, heterodimerization of opioid and CCK receptors subsequent to CCK octapeptide binding was demonstrated. This interaction was identified as the basis of the inhibition of opioid signal transduction and the antagonism of morphine analgesia (Yang et al., 2018; Bernard et al., 2021). CCK antagonists enhance the analgesic efficacy of endogenous opioids in animal models of pain (Inoue, 2017). NPY is pro-analgesic and naloxone administration reduces NPY analgesia (Upadhya et al., 2009). Even though opioids have been very effective in the treatment of pain but in recent years opioid overdose has been a major challenge for the clinical management of pain. Indeed, the most tragic reported drug overdose epidemic in US history was due to opioids. The introduction of a slow-release formulation of oxycodone, in 1996 was found to play a major role in this overdose death or so-called US opioid epidemic (Egilman et al., 2019; Dyer, 2020; Alpert et al., 2021).

## 3 Clinically available drugs

**FIGURE 5:**
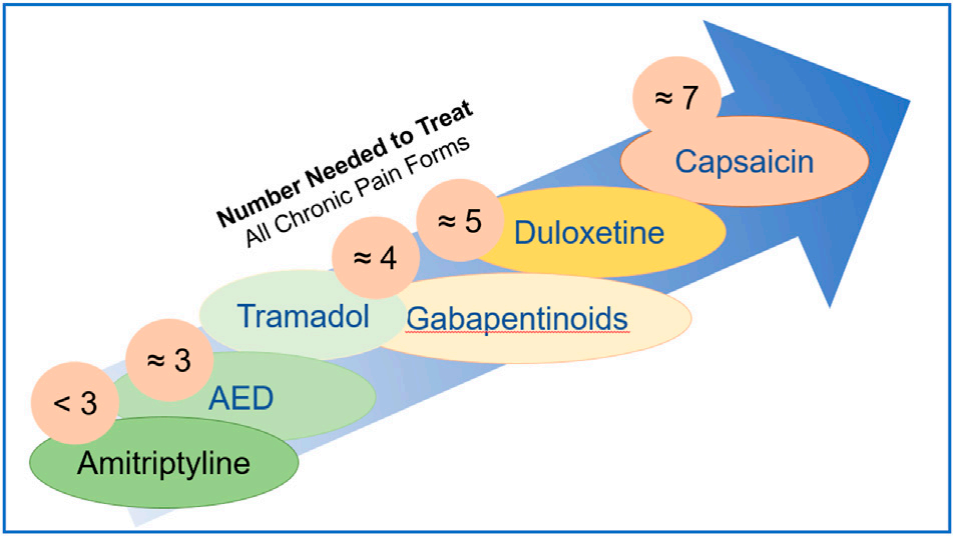
Number needed to treat is defined as the number of patients needed to treat with a certain drug to obtain one patient with a defined degree of pain relief.

The need to estimate and compare drug efficacy for a particular disease is partially satisfied by using NNT (number needed to treat) and NNH (number needed to harm). NNT is defined as the number of patients needed to treat with a certain drug to obtain one patient with a defined degree of pain relief. NNH indicates the number of patients that need to be treated for one patient to drop out due to adverse effects. While the approach works reasonably well for a defined condition, it becomes fuzzy when a multitude of pain causes are amalgamated. Figure 5 shows NNTs for the main drugs used in chronic pain treatment across the different conditions (Finnerup et al., 2005). Table 1 summarizes the sites, modes of action, mechanisms, and examples of drugs used in the relief of pain.

**TABLE 1:** The sites, modes of action, mechanism and examples of drugs used in the relief of pain.

Sites	Modes of action	Mechanism to relief pain	Examples of drugs	Side effects
Transient Receptor Potential (TRPV1, vanilloid subfamily)	Agonist	Deplete substance P and other proalgesic compounds and/or desensitizes receptors	Capsaicin/Resiniferatoxin	Pain, vasodilation/burning sensation
TRP	Ligand	Also acts as an agonist at the cannabinoid receptors	Anandamide	Hypothermia, vasodilation, memory impairment, and immunosuppression
Vesicular nucleotide transporter (VNUT)	Inhibitor	Reduces the ATP concentration and thus its pro-inflammatory effect	Clodronate	Osteonecrosis of the jaw, clinically silent hypocalcemia and gastrointestinal irritation
Purinergic receptor (P2X_4_)	Blocker	Suppress P2X4 receptor-mediated response to ATP and thus its pro-inflammatory effect	Duloxetine/Paroxetine/Maprotiline/Amitriptyline/Clomipramine	Amitriptyline and duloxetine: increase the risk of suicidal thoughts and behaviors, serotonin syndrome, and bleeding. Amitriptyline: anticholinergic effects
	Negative allosteric modulator	Decreases P2X_4_ receptor activity	Alcohol	Cirrhosis, dementia, cardiovascular disease, neuropathy, nutritional deficiencies, and certain cancers
Purinergic receptor (P2X_7_)	Inhibitor of receptor expression	Reduces the expression of spinal P2X7 receptors thus reducing the ATP pro-inflammatory effect	Dexmedetomidine	Bradycardia, hypotension and hypertension
Purinergic receptor (P2X_3_)	Antagonist	Blocks purinergic receptor (P2X_3_)	Gefapixant/Sivopixant	Taste disturbances
Purinergic receptor (P2Y_2_)	Antagonist	Inhibits lowering the activation threshold of TRP channels	Not clinically available	
CD 39 and CD 73 (ectoenzymes)	Positive modulators/activators	Increases the generation of extracellular adenosine (which possesses anti-inflammatory effect)	Not clinically available	
Adenosine kinase and adenosine deaminase	Inhibitor	Decreases the degradation of extracellular adenosine (which possesses anti-inflammatory effect)	Not clinically available	
Adenosine receptor	Blocker	The main inhibitory effect is mediated *via* the adenosine receptor A2A subtype. It also increases plasma adenosine concentration which might activate A3R (which possesses an anti-nociceptive profile)	Caffeine	Improved alertness, cognitive processes, mood, learning and physical performance
Neuronal Voltage Dependent Calcium Channels (VDDC)	Blocker	Reduces calcium channel permeability and calcium cell influx, thus suppressing unspecific neurotransmitter release, including excitatory pro-algetic ones	Gabapentin/Pregabalin	Somnolence and dizziness
Triple monoamine reuptake/calcium and sodium channels	Inhibitor	Inhibits triple monoamine reuptake [NE, 5-HT and DA] thus anti-nociceptive effects. Blocks calcium and sodium channels thus reduction of glutamatergic transmission (anti-hyperalgesic activity)	Nefopam	Drowsiness, sweating and GI disturbances
Serotonin and norepinephrine reuptake	Inhibitor	Inhibits serotonin and norepinephrine reuptake. Its main CYP450 2D6 metabolite is a µ-opioid receptor agonist	Tramadol	Dizziness, somnolence and GI disturbances
Norepinephrine reuptake	Inhibitor	Inhibits norepinephrine reuptake. It is a more potent µ-opioid receptor agonist than tramadol	Tapentadol	Dizziness, somnolence, GI disturbances, dry mouth and pruritis
Sigma 1 receptor (σ1)	Antagonist	Delays the loss of opioid analgesic efficacy. It reduces TRPV1 expression in the plasma membrane of sensory neurons	Ketamine	Psychotomimetic events (vivid dreams) and sialorrhea
5-HT3 receptor	Antagonist	Inhibits the algesia mediated *via* the 5-HT3 receptors. Also, it acts as an agonist of the σ1 receptor	Memantine	Neuropsychiatric adverse effects such as confusion
Phosphodiesterase-1 (PDE I)	Inhibitor	Increases cAMP and thus causes an anti-inflammatory milieu	Amantadine	Constipation, nausea, QT prolongation, orthostatic hypotension, neuropsychiatric symptoms (hallucinations, confusion and delirium) and livedo reticularis
Glutamate transporter (GLT-1)	Upregulation	Enhances glutamate reuptake thus reduction of glutamate-mediated excitation	Ceftriaxone	Clostridioides difficile infection

### 3.1 TRPV1 (vanilloid receptor) acting drugs

**FIGURE 6:**
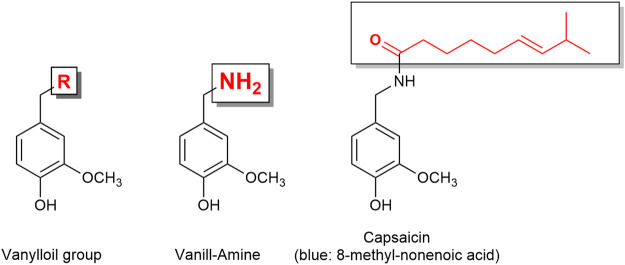
Capsaicin is a vanilloid derivative. The capsaicin receptor was therefore named vanilloid receptor.

The TRP channel superfamily consists of many cation channels divided into six subfamilies. The TRPA1 (ankyrin subfamily, member 1) is known as the wasabi receptor (typical ligand is allyl isothiocyanate, the main pungent compound of wasabi), while the TRPV1 (vanilloid subfamily, member 1) is known as the hot chili pepper receptor [typical ligand is capsaicin (Figure 6), the pungent compound of hot chili pepper]. TRPA1 and TRPV1 have been associated with pain perception (Gouin et al., 2017). Remarkably, it has been reported that almost all sensory neurons expressing TRPA1 (approximately 97%) also express TRPV1 (Story et al., 2003). Functional cross-desensitization has also been reported between the typical agonists of TRPA1 (allyl isothiocyanate; wasabi) and TRPV1 (capsaicin) (Mihara and Shibamoto, 2015). Furthermore, studies have shown that TRPA1 and TRPV1 can form a complex in the plasma membrane, and therefore influence each other’s characteristics (Staruschenko et al., 2010). Thus, Fernandes et al. (2012) described TRPA1 and TRPV1 channels as “partners in crime.”

Ligands at these receptors act from the intracellular side. The best-known activators of TRPV1 are temperature greater than 43°C (109°F), hydrogen ions, capsaicin, allyl isothiocyanate (mustard oil) and resiniferatoxin (Aloum et al., 2021).

Resiniferatoxin (RTX) is a chemical found in some *Euphorbia* species from Africa. Pure RTX is two-to three orders of magnitude hotter than pure capsaicin on the eponymous scale devised by Wilbur Scoville (1865–1942).

Capsaicin has been available as transdermal therapeutic system for many years. On dermal application (8%), it causes pain (dolor) and vasodilation (rubor); many pain specialists advise for use of lidocaine gel/cream prior (1 h) to capsaicin application. Tramadol given well before patch application is considered as an alternative pretreatment option in patients receiving capsaicin patch treatment (Jensen et al., 2014). Resiniferatoxin also causes burning sensation, thus anesthesia or nerve block can be used prior to resiniferatoxin injection (Salas et al., 2017).

Subsequently chronic pain relief sets in with various degrees of efficacy and duration. The assumed mechanism of action is depletion of SP and other proalgesic compounds and/or desensitization of receptors. Overall, the therapeutic approach is less successful than initially hoped for.

### 3.2 Cannabinoid agonist

Anandamide is an endocannabinoid with anxiolytic and hypoalgesic properties (Habib et al., 2019). The name “anandamide” is derived from the Sanskrit word for bliss or delight *ananda*, and amide (Fride and Mechoulam, 1993). In addition to effects mediated *via* cannabinoid receptors, anandamide is also an endogenous ligand (agonist?) for TRP receptors, contributing i.e. to the vasodilation component of inflammation (Russell et al., 2014). Hypothermia, vasodilation, memory impairment, and immunosuppression are side effects of anandamide (Greenberg, 2003) Many other candidates that belong to the cannabinoids family has been used for the management of neuropathic pain as reviewed in (Aviram and Samuelly-Leichtag, 2017; Romero-Sandoval et al., 2017; Bruni et al., 2018; Lee et al., 2018; Mücke et al., 2018; Romero-Sandoval et al., 2018; Boyaji et al., 2020; Meng et al., 2020; Mlost et al., 2020; Urits et al., 2020; Campos et al., 2021). Recently, after the opioid epidemic, attention was diverted to cannabinoids as a therapeutic option for the treatment of pain (Urits et al., 2020).

### 3.3 Vesicular nucleotide transporter (VNUT) inhibitor

TRPV1 activity is subject to regulation by a host of intracellular signaling cascades and extracellular events. As such, modulation of these is a possible and attractive avenue for chronic pain treatment. The fundamental premise as stated by (De Marchi et al., 2019) and other groups is that ATP-induces a pro-inflammatory milieu while an anti-inflammatory environment is adenosine-driven. ATP is released from microglia, keratinocytes, and other cells *via* the activity of the VNUT; inhibition of the transporter reduces the ATP concentration and thus its pro-inflammatory effect mediated *via* interaction with both ionotropic (P2X) and metabotropic (P2Y) purinergic receptors.

**FIGURE 7:**
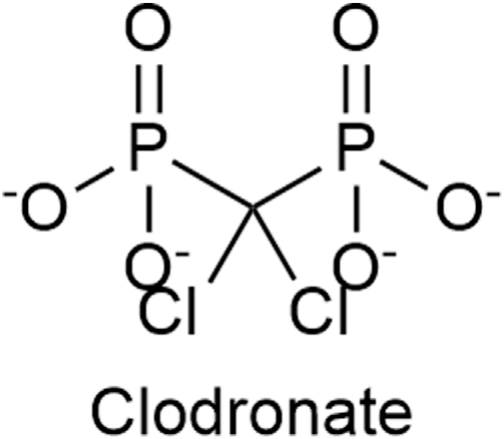
The structure of Clodronate, a first generation non-nitrogen-containing bisphosphonate, which is an inhibitor of the vesicular nucleotide transporter (VNUT).

Clodronate (Figure 7), a first-generation non-nitrogen-containing bisphosphonate, is a potent and selective inhibitor of the VNUT and thus an inhibitor of ATP release. *In vitro* clodronate inhibits VNUT at an IC_50_ ≈ 16 nM without affecting other vesicular neurotransmitter transporters. Clodronate has the potential to suppress inflammatory and neuropathic pain by shifting the balance ATP/adenosine from pro-inflammatory ATP to anti-inflammatory adenosine (Kato et al., 2017; De Marchi et al., 2019; Miras-Portugal et al., 2019; Hasuzawa et al., 2020). Osteonecrosis of the jaw and clinically silent hypocalcemia are side effects associated with the usage of clodronate (Frediani et al., 2018). The gastrointestinal irritation (such as nausea, dyspepsia) is less severe compared to nitrogen-containing bisphosphonates (Suri et al., 2001).

### 3.4 P2X4 receptor blockers

P2X ionotropic purinergic receptors mediate excitatory postsynaptic responses; the main pharmacological distinction between the members of the P2X purinoceptor family is their relative sensitivity to the antagonist suramin (the drug used for the treatment of trypanosomial-induced human sleeping sickness). Seven different P2X receptor subtypes (P2X1-7) have been identified to date; trimeric assembly creates various ionic channels. P2X_4_ receptors have the highest permeability for calcium ions and are thus viewed as most relevant to pain.

P2X4 activation in spinal microglia results in pain hypersensitivity (Inoue, 2017; Stokes et al., 2017; Tozaki-Saitoh et al., 2022).

P2X4 receptor activity is positively modulated by ivermectin, a broad-spectrum anti-parasitic agent included in the WHO essential medicines list for several parasitic diseases (Nörenberg et al., 2012).

**FIGURE 8:**
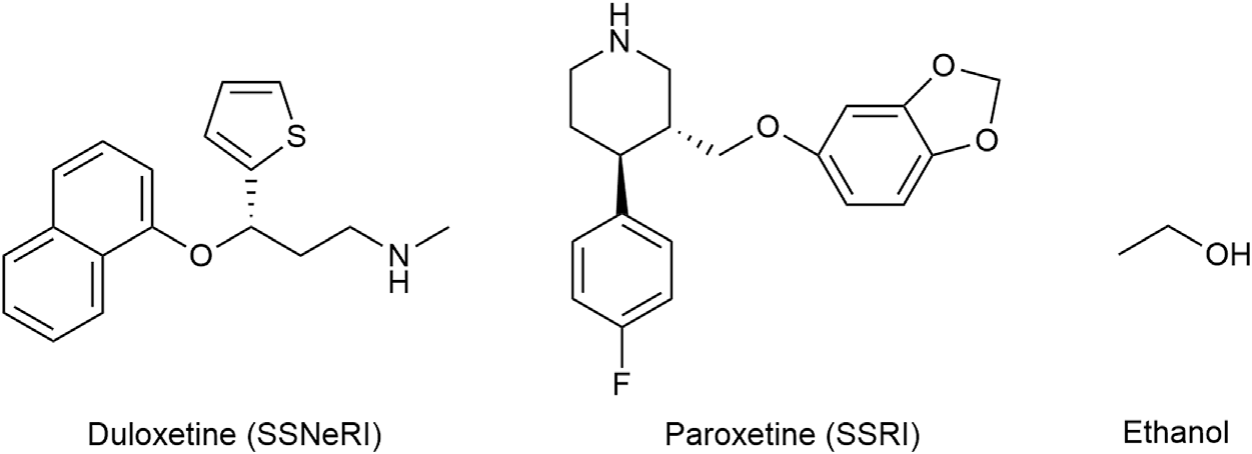
The structures of duloxetine (selective serotonin and norepinephrine reuptake inhibitor—SSNeRI), paroxetine (selective serotonin reuptake inhibitor—SSRI), and ethanol.

The selective serotonin and norepinephrine reuptake inhibitor (SSNeRI) duloxetine (Figure 8) reduces pain *via* augmenting serotonin and norepinephrine mediated inhibitory pain pathways (Smith and Nicholson, 2007). It might alleviate chronic pain also through P2X4 receptor blockade Duloxetine was able to almost completely suppress P2X4 receptor-mediated response to micromolar ATP (Yamashita et al., 2016). The same applies for the selective serotonin reuptake inhibitor (SSRI) paroxetine, and to a lesser extent (≈30% of control) to maprotiline (secondary amine), classical (tertiary amine) tricyclic antidepressants (TCA) (≈50% of control) (amitriptyline and clomipramine) and possibly to most antidepressants used in chronic pain treatment. No effect on P2X4 receptor activity was noticed for mirtazapine and bupropion (Yamashita et al., 2016; Kohno and Tsuda, 2021).

Amitriptyline and duloxetine are recommended first-line treatment for neuropathic pain. Amitriptyline and duloxetine both increase the risk of suicidal thoughts and behaviors, serotonin syndrome, and bleeding; only the former precipitates anticholinergic effects (Bryson and Wilde, 1996; Dougherty et al., 2002; Friedman and Leon, 2007; Leon, 2007; Bartlett, 2017; Bixby et al., 2019; Dhaliwal et al., 2022). The NNT and NNH values are 3.6 (3.0–4.4) and 13.4 (9.3–24.4) for amitriptyline and 6.4 (5.2–8.4) and 11.8 (9.5–15.2) for duloxetine, respectively. Amitriptyline has a selectivity ratio serotonin-to-norepinephrine transporters of approximately 3:1, whereas duloxetine has a 10-fold selectivity for serotonin transporters (Bymaster et al., 2001; Finnerup et al., 2015; Patel and Dickenson, 2022).

The superior NNT for amitriptyline vs. duloxetine contrasts their blocking ability at P2X4 receptor (50% vs. >90%). Several explanations are possible, such as that efficacy for neuropathic pain treatment requires additional mechanisms beyond P2X4 antagonism and that a balanced reuptake inhibition (serotonin to norepinephrine) is of equal, if not greater importance.

While such a line of thinking is perfectly plausible, an alternative explanation is the fact that NNT*s* are estimated against placebo; a highly sedating tertiary amine such as amitriptyline (IC_50_ = 1 nM for the histamine H1 receptor) would have a perceived “beneficiary” effect of facilitating sleep that is difficult to separate from pain reduction. Duloxetine, a non-sedating agent with an IC_50_ = 2,300 nM for the histamine H1 receptor would not have such a benefit (Bymaster et al., 2001). Milnacipran, a non-sedating agent with a selectivity ratio serotonin-to-norepinephrine transporters of approximately 1:3 (mirror values of amitriptyline) has a modest effect in chronic pain with a NNT of only 6 to 10.

The mechanism of action of antidepressants in chronic pain remains highly controversial (Stokes et al., 2017).

Historically ethanol (Figure 8) is one of the oldest analgesics and sedative-hypnotics known to mankind. The molecule being quite promiscuous, the mechanism of action of alcohol is certainly complex. P2X4 are the most ethanol sensitive P2X receptors in the central nervous system and likely mediate some if not the main effect of ethanol on the brain. As the ethanol concentration increases, P2X_4_ activity decreases (Zhang et al., 2020). Alcohol acts as a negative allosteric modulator that blocks the open channel; ivermectin can antagonize the inhibitory effect of ethanol on P2X_4_ and is thought to interfere with ethanol binding to the channel (Kanellopoulos et al., 2021). Chronic alcohol consumption is associated with cirrhosis, dementia, cardiovascular disease (such as hypertension, heart failure, atrial fibrillation), neuropathy, nutritional deficiencies, and certain cancers (Bielinska-Kwapisz and Mielecka-Kubien, 2011; Obad et al., 2018).

### 3.5 P2X_7_ receptor expression inhibitor

Antagonism at P2X7 receptors is assumed to provide multiple benefits (Mishra et al., 2021). Oxidized ATP irreversibly antagonizes P2X7 receptor activation, but lacks selectivity (Murgia et al., 1993; Savio et al., 2018; Rotondo et al., 2022). Brilliant Blue G produced a non-competitive inhibition of human P2X_7_ receptors with IC_50_ values of 200 nM.

**FIGURE 9:**
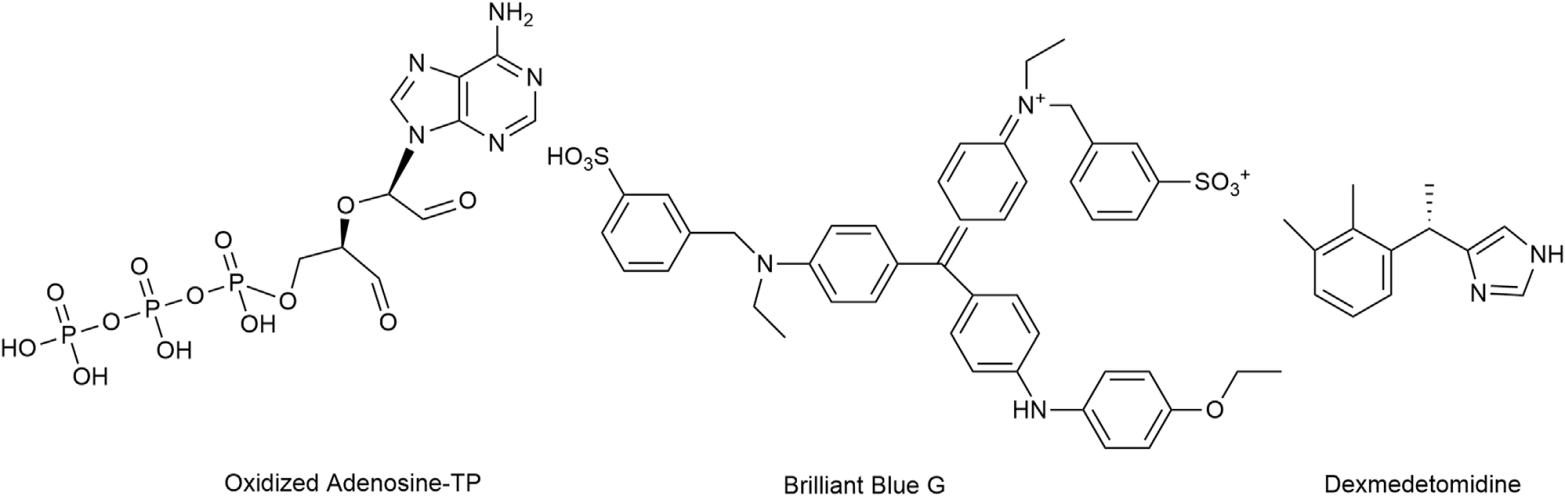
The structures of oxidized Adenosine-TP, Brilliant Blue G and dexmedetomidine, which mediate the inhibition of P2X7 receptors.

While no clinically used compounds interacting selectively/exclusively with P2X7 receptor are available, dexmedetomidine, a centrally acting α2-adrenoceptor agonist used for sedation, was proposed to attenuate neuropathic pain i.a. through inhibition of spinal P2X7 receptor expression (Lin et al., 2018; Zhao et al., 2020) (Figure 9). Bradycardia, hypotension, and hypertension are the most commonly reported side effects (Kaye et al., 2020).

### 3.6 P2X_3_ receptor antagonist

Gefapixant (Merck-7264), structurally similar to trimethoprim, is a P2X3 receptor antagonist named in honour of Geoffrey Burnstock (1929–2020), the father of purinergic pharmacology. Japan authorities approved gefapixant for adults with refractory or unexplained chronic cough. The suffix *-pixant* was adopted for P2X antagonists. Gefapixant has however a low selectivity for P2X_3_ receptors, whereas the newly developed sivopixant has a much higher affinity and selectivity (P2X_3_ IC_50_ ≈ 5 nM). Taste disturbances such as loss or reduced sense of taste are the most reported side effects with gefapixant since P2X_3_ receptors plays an important role in taste perception. Other common side effects include nausea and upper respiratory tract infection. However, lower incidence of taste disturbances is conveyed with sivopixant administration (Abu-Zaid et al., 2021; Niimi et al., 2022).

### 3.7 P2Y_2_ receptor acting drugs

P2Y metabotropic receptors are G-protein-coupled receptors that are stimulated by purine and pyrimidine nucleotides. P2Y1 and/or P2Y2 receptor activation modulates TRP channels by lowering their activation threshold and thus favoring nociception (Tozaki-Saitoh et al., 2022).

Diquafosol, a P2Y_2_ receptor agonist that promotes fluid transfer and mucin secretion by activating receptors expressed on the ocular surface, has been approved in Japan and South Korea to treat dry eye disease (Murakami et al., 2004; Keating, 2015; Nam et al., 2019).

In contrast, an experimental P2Y_2_ receptor-selective antagonist, reversed allodynia in a chronic pain model (Magni et al., 2015). No P2Y_2_ antagonists are clinically available.

### 3.8 CD 39 and CD 73 positive modulator

CD 39 (EC 3.6.1.5) and CD 73 (EC 3.1.3.5) are ectoenzymes involved in the production of AMP from phosphorylated precursors (CD 39) and adenosine from AMP (CD 73) (Liu et al., 2017). They participate in regulating inflammatory processes and in nociceptive modulation by affecting extracellular adenosine generation.

Cell surface (ecto)nucleotidases serving to terminate purinergic signaling are seen as analogous to the activity of cholinesterases at cholinergic synapses (Stokes et al., 2017). Manipulating extracellular AMP hydrolysis could provide an alternative mechanism to control pain (Zylka, 2011; Liu et al., 2017).

Inhibiting overexpressed ectoenzymes involved in the production of adenosine in cancer cells may improve outcomes of conventional cancer therapy by decreasing adenosine levels and, consequently, promoting antitumor immune cells (Azambuja et al., 2019; Battastini et al., 2021).

Conversely, positive modulators/activators of ectoenzymes, are of potential interest in reducing/modulating pain, contingent on our ability to control off-target effects (Sowa et al., 2010). No positive modulators or antagonists are to date clinically available.

### 3.9 Adenosine kinase (EC 2.7.1.20) and adenosine deaminase (EC 3.5.4.4) inhibitors

Adenosine is rapidly removed from the extracellular space by nucleoside transporters and metabolic enzymes, including adenosine kinase (intracellular conversion to AMP and trapping) and adenosine deaminase (conversion to inosine). Indeed, the extracellular concentration of adenosine and the antinociceptive effects of adenosine can be increased by pharmacologically inhibiting these metabolic enzymes (Sowa et al., 2010; Pastor-Anglada and Pérez-Torras, 2018).

Clinical development of adenosine kinase inhibitors was stopped due to toxicity (Boison and Jarvis, 2021).

### 3.10 Adenosine receptor blocker

**FIGURE 10:**
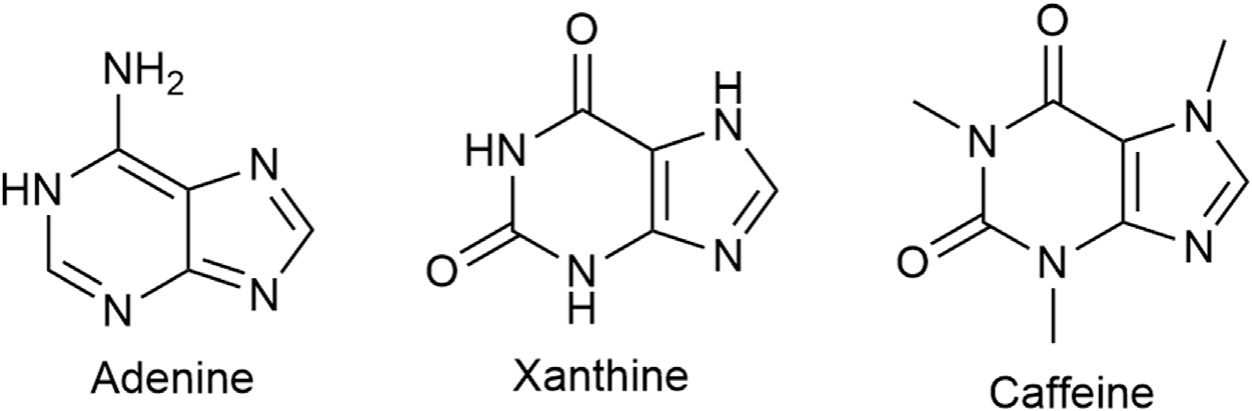
Caffeine, a xanthine derivative, is a non-selective adenosine receptor blocker. Adenine is a precursor of xanthine and adenine nucleotide is a precursor of adenosine.

Caffeine (Figure 10), a tri-methylated (1; 3;7) xanthine derivative and the most consumed psychoactive drug worldwide, has putative analgesic and/or anti-nociceptive effects. Its inclusion as an adjuvant in a number of double combination preparations, mostly with an NSAID (ibuprofen, naproxen, acetylsalicylic acid) or acetaminophen and triple combinations with an NSAID and acetaminophen modestly lowers the NNT (the lower the NNT the more efficacious the drug is) (Petersen, 2013; Yancey and Dattoli, 2013). The usual caffeine dose in combination preparations is 65 mg with acetaminophen and 100 mg with ibuprofen. For the general population of healthy adults, Health Canada advises a daily intake of no more than 400 mg.

The exact mechanism(s) of analgesic and/or anti-nociceptive effect(s) of caffeine is/are not known.

Caffeine is a non-selective adenosine receptor (AR) blocker. The affinity for the AR subtypes varies. Researchers from the Swiss Federal Institute of Technology in Lausanne report affinity values of caffeine for the human adenosine receptors as 12 at A1, 2.4 at A2A, 13 at A2B, and 80 μM at A3 (Froestl et al., 2012).

Administration of 160 mg caffeine (≈800 μM), the rough equivalent of one cup of coffee, generates mean maximal plasma concentrations of 18 μM (White et al., 2016). Administration of 100 mg caffeine (≈500 μM) generates mean maximal plasma concentrations of 15 μM (Grzegorzewski et al., 2021). Caffeine concentration in brain structures is about 50% of caffeine level in plasma (Orrego et al., 2016).

With estimated maximal CNS concentrations of caffeine at 10 μM, one can assume that the main inhibitory effect is mediated *via* A2A receptors; some inhibition of A1 and A2B receptors is possible, while a direct effect at A3 receptors appears unlikely.

A2A have anti-inflammatory effects and modify pain by direct and indirect actions (Sawynok, 2016). A2AR agonists however show some peripheral pro-nociceptive effects (Jung et al., 2022).

Furthermore, caffeine significantly increases plasma adenosine concentration (Conlay et al., 1997). As such, a shift of adenosine effect from caffeine blocked receptors to the unaffected A3R appears possible. A3R have an anti-nociceptive profile *via* actions on spinal microglia (Sawynok, 2016). Several experimental A3R agonists are being considered as promising analgesic agents (Jung et al., 2022).

Adding further complexity is the propensity of adenosine receptors to form homo-and hetero-dimers such as A1-A2A. In a must-read review titled “Pharmacology of Adenosine Receptors: The State of the Art” the authors point out that: *“Low adenosine concentration preferentially stimulates the A1 protomer of the heteromer, which would inhibit glutamatergic transmission. On the other hand, high adenosine concentration activates adenosine A2A protomer of the heteromer that blocks adenosine A1-mediated effects and results in potentiation of glutamate release”* (Borea et al., 2018). No information concerning the interaction between caffeine and A1-A2A adenosine receptor dimers is available to us.

In addition, caffeine is also an antagonist at the inositol trisphosphate (IP_3_)- receptors subtype 1. High concentrations of caffeine (10–70 mM) selectively inhibit IP_3_-R1 without affecting IP_3_ binding and oppose the intracellular Ca^2+^ increase (Saleem et al., 2014). The caffeine concentrations required for IP_3_-R1 inhibition are in the toxic range and therefore clinically not relevant.

Same applies to the ryanodine receptor where low mM (5 mM) concentrations of caffeine activate the receptor.

Caffeine is also a weak blocker of the ionotropic-glycine receptor (GlyR). Docking simulations indicate that caffeine and strychnine may bind to similar sites at the GlyR. Tested against the EC_50_ of each GlyR subtype, the inhibitory caffeine concentration (IC_50_) is in the range of 200–1,000 μM. GlyR produces its inhibitory effects through hyperpolarizing chloride currents (Duan et al., 2009). The caffeine concentrations required for GlyR inhibition are in the toxic range and therefore clinically not relevant for analgesia.

In conclusion, the analgesic/anti-nociceptive mechanism of action of caffeine is unclear. However, the effects are more established including improved alertness, cognitive processes, mood, learning and physical performance (Wikoff et al., 2017; Soós et al., 2021).

### 3.11 Gabapentinoids: Gabapentin and pregabalin

**FIGURE 11:**
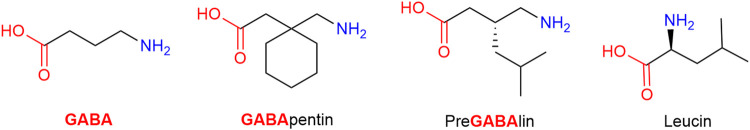
Structurally gabapentin and pregabalin are related to the endogenous inhibitory neurotransmitter GABA and to the amino acid leucine.

Gabapentin and pregabalin are structurally related to the endogenous inhibitory neurotransmitter GABA and to the amino acids leucine and isoleucine; in fact, both gabapentin and pregabalin contain GABA in both name and structure (Figure 11). Despite the similarity, their affinity for GABA receptors is orders of magnitude lower (high Ki) than for the α2δ auxiliary subunit of neuronal Voltage Dependent Calcium Channels (VDDC), which they bind to and block. By doing so, calcium channel permeability and calcium cell influx are reduced, leading subsequently to an unspecific neurotransmitter release reduction, including excitatory pro-algetic ones (Lanzetti and Di Biase, 2022). It is assumed that the endogenous agonists at the α2δ auxiliary subunit are the essential, branched-chain amino acids leucine and isoleucine, and thus gabapentin and pregabalin function as leucine/isoleucine competitive antagonists. While both drugs are recommended as possible first-line treatment for neuropathic pain by various medical authorities, the NNT*s* are high. Generally, the consensus is that tertiary amine tricyclic antidepressants (TA-TCA; amitriptyline) have superior (low) NNT values, but gabapentinoids are safer drugs (Finnerup et al., 2005). Stated more bluntly, it is easy and often infaust to overdose on TA-TCA, while a similar outcome would require enthusiastic use of gabapentinoids and still not offer any guarantee (Montgomery et al., 1989; Fischer et al., 1994). However, both medications share the most frequently reported adverse effects, which are somnolence and dizziness (Baidya et al., 2011; Chang et al., 2014).

### 3.12 Other drugs

#### 3.12.1 Nefopam

**FIGURE 12:**
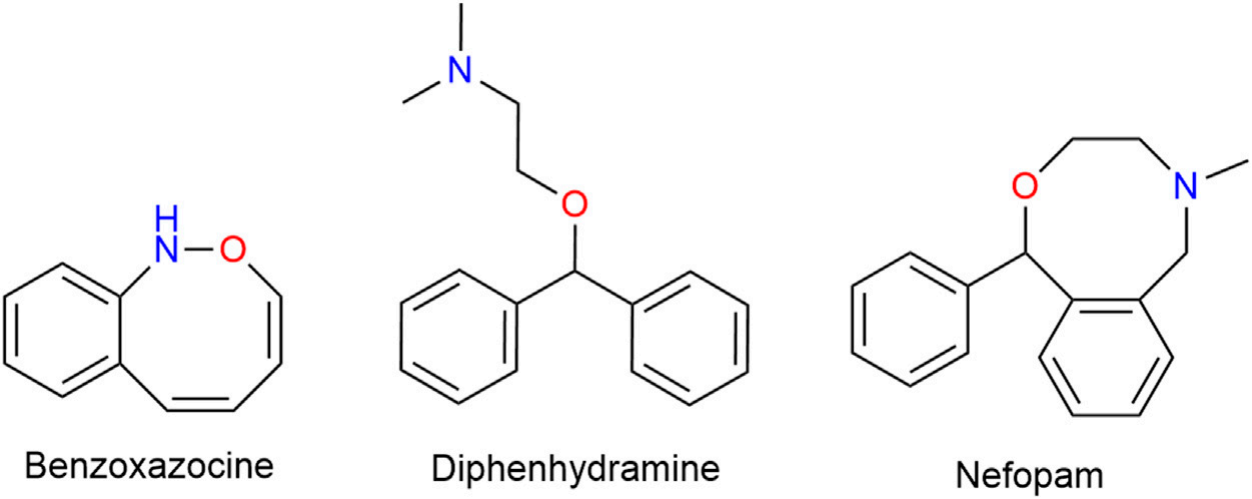
Structurally a benzoxazocine, nefopam is related to diphenhydramine.

Nefopam (NFP) is a non-opioid, non-steroidal, centrally acting analgesic drug that does not inhibit PG synthesis. Structurally a benz-oxazocine, NFP is related to orphenadrine and diphen-hydramine. It has anti-cholinergic but no anti-histaminergic side-effects (Figure 12) (Heel et al., 1980).

NFP acts at spinal and supra spinal sites and exerts anti-nociceptive effects through triple monoamine reuptake inhibition [NE, 5-HT and DA], and anti-hyperalgesic activity through blockade of calcium and sodium channels and subsequent reduction of glutamatergic transmission (Alfonsi et al., 2004; Kim and Abdi, 2014; Girard et al., 2016). NFP is also effective for prevention of intra- and post-anesthetic shivering (meperidine/pethidine-like effect). The substance has approximately half of the analgesic potency of morphine but does not produce respiratory depression (Piper et al., 1998; Piper et al., 2004). NFP overdose causes neurological impairment such as disorientation, seizure, and cardiovascular and anticholinergic effects. However, NFP is generally well-tolerated, in which the reported side effects are minor for example drowsiness, sweating and GI disturbances (Park et al., 2014; Jeon et al., 2019). Based on NFP’s mechanisms of analgesic action, it is more suitable for the treatment of neuropathic pain (Kim and Abdi, 2014). The 5-HT_7_ receptor (but not 5-HT_3_) is involved in the antiallodynic action of NFP in the spinal cord (Dam et al., 2014; Lee et al., 2015).

#### 3.12.2 Tramadol

**FIGURE 13:**
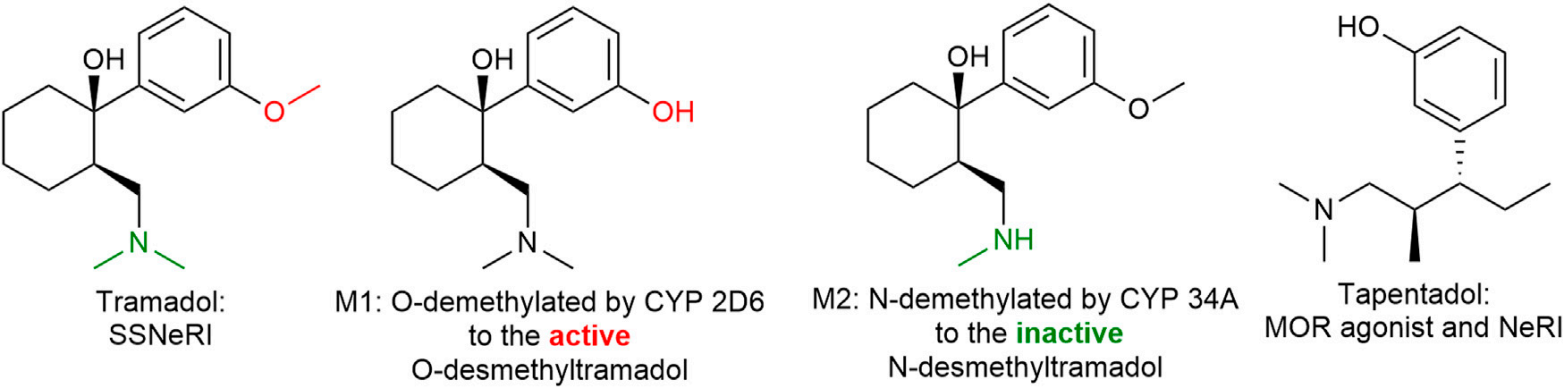
Tramadol and tapentadol are similar in their inhibition of norepinephrine reuptake. Tapentadol is also a more potent µ-opioid receptor agonist than tramadol. SSNeRI: selective serotonin and norepinephrine reuptake inhibitor, MOR: µ-opioid receptor, and NeRI: norepinephrine reuptake inhibitor.

Tramadol (Figure 13) is a familiar drug to physicians, having been on the European market for over 50 years and its pharmacodynamic actions are well understood. The (+) enantiomer preferentially inhibits serotonin reuptake and enhances serotonin efflux in the brain, whereas the (−) enantiomer mainly inhibits noradrenaline reuptake thus the racemic mixture itself is a balanced (equal serotonin and norepinephrine) reuptake inhibitor. Its main CYP450 2D6 metabolite (M1: ODM-tramadol) is an µ-opioid receptor agonist with poor blood-brain barrier permeability (Fudin and Boglish, 2016). The opioid mediated analgesic efficacy of tramadol (after metabolic conversion) is about 1/10 of that of morphine (comparable with codeine) and dependent on the activity of the highly polymorphic cytochrome P450 enzyme 2D6 (Gan et al., 2007).

As an intravenous drug it was (and still is) used for acute pain on ambulances and in hospital with various degrees of success due to the slow onset of action and variable efficacy. More established is tramadol’s use orally for chronic pain (Fudin and Boglish, 2016; Subedi et al., 2019). In addition, it is preferred over NSAIDs due to the better tolerability as tramadol common adverse effects include dizziness, somnolence and GI disturbances while NSAIDs can cause renal and GI impairment. The low addiction rate also makes tramadol a preferable choice for analgesia in comparison to other opioid medications (Vazzana et al., 2015).

#### 3.12.3 Tapentadol

Tapentadol (Figure 13), is similar to (−)tramadol in as much as it inhibits mainly norepinephrine reuptake. It is a more potent µ-opioid receptor agonist than tramadol (in fact tramadol is only a partial agonist) and does not require hepatic activation, thus having a more predictable and faster onset of action. The side effect profile is similar to tramadol and other opioids such as GI disturbances, somnolence, dizziness, dry mouth, and pruritis (Singh et al., 2013). Tapentadol might possess a lower risk of nausea, vomiting, and hypoglycemia however a greater risk of respiratory depression (yet rare), constipation, and overdose complications than tramadol (Roulet et al., 2021).

#### 3.12.4 Ketamine

**FIGURE 14:**
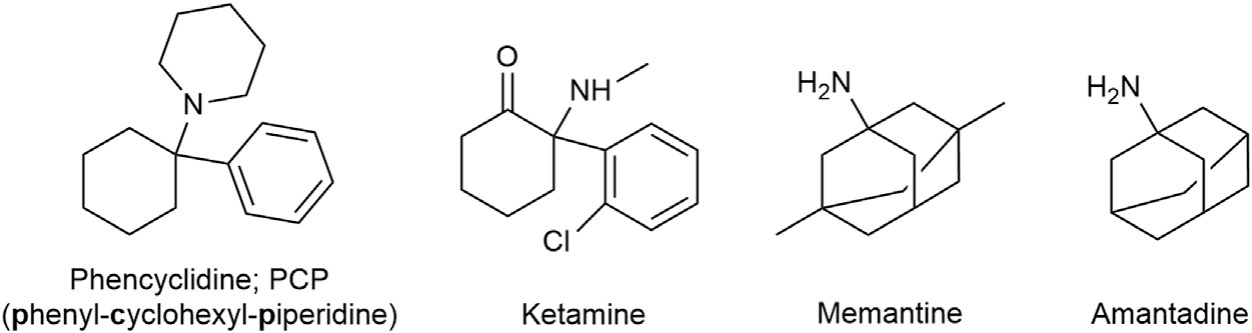
Ketamine is related to Phencyclidine. Memantine is an amantadine derivative.

Ketamine is a familiar drug to most emergency and trauma physicians, having been clinically available for over half a century. Since the Vietnam War ketamine is the standard analgesic/anesthetic for mass casualties and out-of-hospital trauma emergencies. Chemically an arylcyclohexylamine derivative related to phencyclidine (phenyl-cyclohexyl-piperidine; PCP), ketamine (Figure 14) is a promiscuous drug interacting with a large number of receptors (Diwo and Petroianu, 2002). The main mechanism of anesthetic action is NMDA receptor antagonism (Ki ≈ 200–500 nM) translating in a unique state of *dissociation* (dissociative anesthesia) assumed to be due to interruption of the thalamo-cortical pathway. The major advantage of ketamine anesthesia is maintenance of respiratory drive and cardiac output, associated with sympathetic nervous system activation. On the negative side there is a relatively high incidence of psychotomimetic events (vivid dreams) and sialorrhea, the later likely due to muscarinic M3 receptor activation. Co-administration of a benzodiazepine and a muscarinic antagonist reduces these side-effects.

The role of subanesthetic doses of ketamine in the treatment of neuropathic and chronic pain is less well defined. Also less understood is the site and mechanism of action of ketamine in neuropathic/chronic pain, most likely involving actions at additional receptors [sigma 1 (σ1) receptor] and efferent inhibitory pain pathways (Niesters et al., 2014). The σ1 ligands that enhance µ-opioid receptor (MOR) analgesia are referred to as antagonists, and those that reduce opioid analgesia and/or oppose the effects of antagonists are classified as agonists. The antagonistic activity of ketamine at σ1 receptors translates clinically in delayed loss of opioid analgesic efficacy; the σ1 receptor and the MOR co-regulate the activity of many TRP calcium channels (Cortés-Montero et al., 2019). This is the theoretical basis for co-administration of ketamine with an opiate. In addition, σ1 antagonists, alter or reduce TRPV1 expression in the plasma membrane of sensory neurons (Ortíz-Rentería et al., 2018).

Sanacora and Schatzberg (2015) in their review speak of a *web of glutamatergic confusion*.

#### 3.12.5 Memantine

Memantine (Figure 14), an amantadine derivative marketed for Alzheimer dementia as “*the better magnesium,*” is another promiscuous NMDA antagonist. The Ki-value of memantine at the phencyclidine binding site of the NMDA receptor is similar to that of ketamine (Kornhuber et al., 1989). In contrast to ketamine however, memantine is not trapped in the cationic channel and can rapidly dissociate out of the protein (*rapid roll in - rapid roll off*). Memantine also shows antagonist effects at the 5-HT3 receptor, with an affinity similar to that for the NMDA receptor. 5-HT3 receptor activation is assumed to favor algesia. Memantine (and amantadine; see below) also bind to and act as agonists (with comparable affinities) of the σ1 receptor. The drug was used for neuropathic/chronic pain treatment, the data is however very limited (Sanacora and Schatzberg, 2015; Nair and Sahoo, 2019). Memantine is generally well-tolerated with no serious side effects however high dosages can precipitate neuropsychiatric adverse effects such as confusion (Kornhuber et al., 2007; Nair and Sahoo, 2019).

#### 3.12.6 Amantadine

Eisenberg and Pud (1998) asked *Can patients with chronic neuropathic pain be cured by acute administration of the NMDA receptor antagonist amantadine?*

Amantadine has been suggested to inhibit NMDA receptors by accelerating the channel closing, in turn leading to stabilization of the channel in the closed state (Blanpied et al., 2005). Recently it was questioned whether NMDA receptors are major targets for the therapeutic activity of amantadine. (Danysz et al., 2021). The authors indicate that at therapeutically achievable concentrations the most likely targets are intra-cellular proteins: phosphodiesterase-1 (PDE I), aromatic amino acids decarboxylase (AADC) and the sigma 1 receptor (σ1R).

PDE I inhibition translates in a cAMP increase and thus possibly in an anti-inflammatory milieu (O'Brien et al., 2020). Regardless of the actual mechanism of action therapeutic success in pain control appears to be limited (Aiyer et al., 2018). A range of side effects have been reported with the usage of amantadine including constipation, nausea, QT prolongation, orthostatic hypotension, neuropsychiatric symptoms (hallucinations, confusion and delirium) and livedo reticularis (Perez-Lloret and Rascol, 2018).

#### 3.12.7 Magnesium

Magnesium, the endogenous NMDA antagonist, is assumed to play an important role in the prevention of central sensitization and in the attenuation of established pain hypersensitivity. The drug was used for neuropathic/chronic pain treatment, the supportive data is however limited (Na et al., 2011; Shin et al., 2020; Morel et al., 2021).

#### 3.12.8 Ceftriaxone

Several lines of evidence suggest analgesic effects of the third-generation parenteral cephalosporin antibiotic ceftriaxone in preclinical pain models. The assumed mechanism of action is enhancement of the glutamate reuptake by upregulation of the glutamate transporter GLT-1 in the spinal cord and elsewhere (Sisignano et al., 2022). GLT-1 is a sodium-dependent transporter that plays a key role in glutamate homeostasis by removing excess glutamate in the central nervous system (Peterson and Binder, 2019). Due to GLT-1 upregulation, glutamate concentration and glutamate mediated excitation decrease. The glial glutamate transporter is referred to as GLT-1 in the rodent literature and excitatory amino acid transporter 2 (EAAT2) in the human literature (Wikipedia, 2022). Ceftriaxone share similar side effect to other cephalosporins including clostridioides difficile infection (Hensgens et al., 2012).

### 3.13 Antibodies

#### 3.13.1 Anti-CGRP antibodies

While CGRP receptor inhibitors (-gepant) are mainly used for the treatment of migraine attacks, monoclonal antibodies targeting either CGRP receptor or ligand are effective in the prevention of migraine attacks (Edvinsson, 2021). FDA approved anti-CGRP antibodies erenumab, fremanezumab, and galcanezumab in 2018 and eptinezumab in 2020 for the prophylactic treatment in chronic migraine (Schwedt and Garza, 2022). The stimulation of trigeminal ganglion causes CGRP release; CGRP concentrations in jugular blood were found to be elevated during migraine attacks (Goadsby and Edvinsson, 1993). The monoclonal antibodies against the neuropeptide CGRP act *via* inhibition of neurogenic vasodilation in the dura and skin. Anti-CGRP antibodies are superior to other treatments due to their high specificity for the target, limited side effects and toxicities and long duration of action (Charles and Pozo-Rosich, 2019; Edvinsson, 2021).

#### 3.13.2 Nerve growth factor antibodies

Nerve growth factor (NGF), secreted following nerve injury, binds to its receptor and activates MAPKs and nuclear factor-kappa B (NF-κB) signaling pathways. This mediates the increase and sensitization of several substances such as substance P, CGRP, brain-derived neurotrophic factor (BDNF) and TRPV1 thus modulating neuropathic pain (Dai et al., 2020). Several studies showed that NGF concentration is elevated in chronic pain such as lumbar disc herniation, osteoarthritis, and low back pain (Onda et al., 2005; Orita et al., 2011; Shi et al., 2018). Thus, antibodies targeting NGF including tanezumab, fulranumab, and fasinumab have been developed and tested in clinical trials, however unfortunately FDA stopped all trials related to anti-NGF antibodies due to reported fast joint destruction (Bannwarth and Kostine, 2014).

## 4 Future possible therapeutics

Neuropathic pain is a major clinical problem; unfortunately, we are still not able to adequately relief pain. Several other pathways have been found to be involved in pain mechanism and thus are potential future drug targets. The *α*-Subunit of the voltage-gated sodium channel, Na_v_1.7, is a key player in the transmission of pain signals; it evokes an action potential following depolarization of neurons by harmful stimuli (Minett et al., 2012). Mutations in SCN9A gene encoding for Na_v_1.7 are associated with pain disorders such primary hereditary erythromelalgia and paroxysmal extreme pain disorder (Yang et al., 2004; Drenth et al., 2005; Fertleman et al., 2006; Sheets et al., 2007). On the contrary, mutations involving loss of function of Na_v_1.7 causes severe impairment of pain perception known as congenital indifference to pain (Cox et al., 2006; Goldberg et al., 2007). Hence, Na_v_1.7-targeted blockers are potential analgesics; the combination with opioids produced analgesia in animals (Emery et al., 2016).

In neuropathic pain, loss of Potassium-Chloride Cotransporter (KCC2) functional expression in spinal cord dorsal horn neurons contributes to GABAergic disinhibition. KCC2 expels chloride ions out of the neurons and hence guarantees a low intracellular chloride concentration, which is needed for proper GABAergic transmission. The activation of GABA-A receptors causes an influx of chloride and ultimately hyperpolarization. When KCC2 activity is reduced, chloride will accumulate in the neuron and thus less hyperpolarization will occur upon GABA-A receptor activation, causing neuropathic pain. Hence, KCC2 enhancers constitute another potential new therapeutic strategy to reduce neuropathic pain (Kahle et al., 2014). Gagnon et al. (2013) were successful in finding a KCC2 analogue (CLP257); this agonist enhanced the extrusion of chloride and thus caused hyperpolarization in spinal neurons of a rat model of neuropathic pain. In addition, kenpaullone is another successful example of KCC2 modulator. It improved *Kcc2/KCC2* gene expression by negatively affecting the function of glycogen synthase kinase-3 (GSK3ß). Kenpaullone was found to be an effective analgesic in mouse models of pathologic pain (Yeo et al., 2021). Therefore, many future directions are yet to be explored from classic receptor modulation to novel genetic reprogramming.

## 5 Conclusion

Acute pain is a protective physiological reaction to noxious stimuli; in contrast chronic pain is pain that lost most or any usefulness. Treatment of chronic pain is difficult and often frustrating -both for patient and physician-as symptomatic treatment so often is. The symptom of chronic pain is the common final pathway for many conditions of most various etiology. It is not surprising that therapeutic successes are limited. Understanding the multitude of possible molecular mechanisms generating the common symptom pain is a highly desirable but a faraway goal. Familiarity with the pharmacodynamic action of the available analgesic drugs allows drug combinations with complementary, possibly synergistic actions, and non-additive adverse reactions.

## References

[B1] Abu-Zaid A. Aljaili A. K. Althaqib A. Adem F. Alhalal D. A. Almubarak A. F. (2021). Safety and efficacy of gefapixant, a novel drug for the treatment of chronic cough: A systematic review and meta-analysis of randomized controlled trials. Ann. Thorac. Med. 16 (2), 127–140. 10.4103/atm.ATM_417_20 34012479 PMC8109686

[B2] Aiyer R. Mehta N. Gungor S. Gulati A. (2018). A systematic review of NMDA receptor antagonists for treatment of neuropathic pain in clinical practice. Clin. J. Pain 34 (5), 450–467. 10.1097/ajp.0000000000000547 28877137

[B3] Alfonsi P. Adam F. Passard A. Guignard B. Sessler D. I. Chauvin M. (2004). Nefopam, a nonsedative benzoxazocine analgesic, selectively reduces the shivering threshold in unanesthetized subjects. Anesthesiology 100 (1), 37–43. 10.1097/00000542-200401000-00010 14695722 PMC1283107

[B4] Aloum L. Alefishat E. Shaya J. Petroianu G. A. (2021). Remedia sternutatoria over the centuries: TRP mediation. Molecules 26 (6), 1627. 10.3390/molecules26061627 33804078 PMC7998681

[B5] Alpert A. Evans W. N. Lieber E. M. J. Powell D. (2021). Origins of the opioid crisis and its enduring impacts. Q. J. Econ. 137 (2), 1139–1179. 10.1093/qje/qjab043 35832727 PMC9272388

[B6] Aviram J. Samuelly-Leichtag G. (2017). Efficacy of cannabis-based medicines for pain management: A systematic review and meta-analysis of randomized controlled trials. Pain Physician 20 (6), E755–E796. 10.36076/ppj.20.5.e755 28934780

[B7] Azambuja J. H. Ludwig N. Braganhol E. Whiteside T. L. (2019). Inhibition of the adenosinergic pathway in cancer rejuvenates innate and adaptive immunity. Int. J. Mol. Sci. 20 (22), 5698. 10.3390/ijms20225698 31739402 PMC6888217

[B8] Baidya D. K. Agarwal A. Khanna P. Arora M. K. (2011). Pregabalin in acute and chronic pain. J. Anaesthesiol. Clin. Pharmacol. 27 (3), 307–314. 10.4103/0970-9185.83672 21897498 PMC3161452

[B9] Bannwarth B. Kostine M. (2014). Targeting nerve growth factor (NGF) for pain management: What does the future hold for NGF antagonists? Drugs 74 (6), 619–626. 10.1007/s40265-014-0208-6 24691709

[B10] Bartlett D. (2017). Drug-induced serotonin syndrome. Crit. Care Nurse 37 (1), 49–54. 10.4037/ccn2017169 28148614

[B11] Battastini A. M. O. Figueiró F. Leal D. B. R. Doleski P. H. Schetinger M. R. C. (2021). CD39 and CD73 as promising therapeutic targets: What could Be the limitations? Front. Pharmacol. 12, 633603. 10.3389/fphar.2021.633603 33815115 PMC8014611

[B12] Bernard A. Danigo A. Bourthoumieu S. Mroué M. Desmoulière A. Sturtz F. (2021). The cholecystokinin type 2 receptor, a pharmacological target for pain management. Pharm. (Basel) 14 (11), 1185. 10.3390/ph14111185 PMC8618735 34832967

[B13] Bielinska-Kwapisz A. Mielecka-Kubien Z. (2011). Alcohol consumption and its adverse effects in Poland in years 1950–2005. Econ. Res. Int. 2011, 1–13. 10.1155/2011/870714

[B14] Bixby A. L. VandenBerg A. Bostwick J. R. (2019). Clinical management of bleeding risk with antidepressants. Ann. Pharmacother. 53 (2), 186–194. 10.1177/1060028018794005 30081645

[B15] Blanpied T. A. Clarke R. J. Johnson J. W. (2005). Amantadine inhibits NMDA receptors by accelerating channel closure during channel block. J. Neurosci. 25 (13), 3312–3322. 10.1523/jneurosci.4262-04.2005 15800186 PMC6724906

[B16] Boison D. Jarvis M. F. (2021). Adenosine kinase: A key regulator of purinergic physiology. Biochem. Pharmacol. 187, 114321. 10.1016/j.bcp.2020.114321 33161022 PMC8096637

[B17] Borea P. A. Gessi S. Merighi S. Vincenzi F. Varani K. (2018). Pharmacology of adenosine receptors: The state of the Art. Physiol. Rev. 98 (3), 1591–1625. 10.1152/physrev.00049.2017 29848236

[B18] Bowers M. E. Choi D. C. Ressler K. J. (2012). Neuropeptide regulation of fear and anxiety: Implications of cholecystokinin, endogenous opioids, and neuropeptide Y. Physiol. Behav. 107 (5), 699–710. 10.1016/j.physbeh.2012.03.004 22429904 PMC3532931

[B19] Boyaji S. Merkow J. Elman R. N. M. Kaye A. D. Yong R. J. Urman R. D. (2020). The role of cannabidiol (CBD) in chronic pain management: An assessment of current evidence. Curr. Pain Headache Rep. 24 (2), 4. 10.1007/s11916-020-0835-4 31980957

[B20] Brenchat A. Romero L. García M. Pujol M. Burgueño J. Torrens A. (2009). 5-HT7 receptor activation inhibits mechanical hypersensitivity secondary to capsaicin sensitization in mice. Pain 141 (3), 239–247. 10.1016/j.pain.2008.11.009 19118950

[B21] Bruni N. Della Pepa C. Oliaro-Bosso S. Pessione E. Gastaldi D. Dosio F. (2018). Cannabinoid delivery systems for pain and inflammation treatment. Molecules 23 (10), 2478. 10.3390/molecules23102478 30262735 PMC6222489

[B22] Bryson H. M. Wilde M. I. (1996). Amitriptyline. A review of its pharmacological properties and therapeutic use in chronic pain states. Amitriptyline. Drugs & Aging 8 (6), 459–476. 10.2165/00002512-199608060-00008 8736630

[B23] Bymaster F. P. Dreshfield-Ahmad L. J. Threlkeld P. G. Shaw J. L. Thompson L. Nelson D. L. (2001). Comparative affinity of duloxetine and venlafaxine for serotonin and norepinephrine transporters *in vitro* and *in vivo*, human serotonin receptor subtypes, and other neuronal receptors. Neuropsychopharmacology 25 (6), 871–880. 10.1016/s0893-133x(01)00298-6 11750180

[B24] Campos R. M. P. Aguiar A. F. L. Paes-Colli Y. Trindade P. M. P. Ferreira B. K. de Melo Reis R. A. (2021). Cannabinoid therapeutics in chronic neuropathic pain: From animal research to human treatment. Front. Physiol. 12, 785176. 10.3389/fphys.2021.785176 34916962 PMC8669747

[B25] Chang C. Y. Challa C. K. Shah J. Eloy J. D. (2014). Gabapentin in acute postoperative pain management. BioMed Res. Int. 2014, 631756. 10.1155/2014/631756 24829909 PMC4009126

[B26] Charles A. Pozo-Rosich P. (2019). Targeting calcitonin gene-related peptide: A new era in migraine therapy. Lancet 394 (10210), 1765–1774. 10.1016/s0140-6736(19)32504-8 31668411

[B27] Cohen S. P. Mao J. (2014). Neuropathic pain: Mechanisms and their clinical implications. Bmj 348, f7656. 10.1136/bmj.f7656 24500412

[B28] Conlay L. A. Conant J. A. deBros F. Wurtman R. (1997). Caffeine alters plasma adenosine levels. Nature 389 (6647), 136. 10.1038/38160 9296490

[B29] Cortés-Montero E. Sánchez-Blázquez P. Onetti Y. Merlos M. Garzón J. (2019). Ligands exert biased activity to regulate sigma 1 receptor interactions with cationic TRPA1, TRPV1, and TRPM8 channels. Front. Pharmacol. 10, 634. 10.3389/fphar.2019.00634 31249525 PMC6582314

[B30] Cox J. J. Reimann F. Nicholas A. K. Thornton G. Roberts E. Springell K. (2006). An SCN9A channelopathy causes congenital inability to experience pain. Nature 444 (7121), 894–898. 10.1038/nature05413 17167479 PMC7212082

[B31] Dai W.-L. Yan B. Bao Y.-N. Fan J.-F. Liu J.-H. (2020). Suppression of peripheral NGF attenuates neuropathic pain induced by chronic constriction injury through the TAK1-MAPK/NF-κB signaling pathways. Cell Commun. Signal. 18 (1), 66. 10.1186/s12964-020-00556-3 32312253 PMC7171864

[B32] Dam L. J. Hai L. Ha Y. M. (2014). Role of the 5-HT(7) receptor in the effects of intrathecal nefopam in neuropathic pain in rats. Neurosci. Lett. 566, 50–54. 10.1016/j.neulet.2014.02.021 24561091

[B33] Danysz W. Dekundy A. Scheschonka A. Riederer P. (2021). Amantadine: Reappraisal of the timeless diamond-target updates and novel therapeutic potentials. J. Neural Transm. (Vienna) 128 (2), 127–169. 10.1007/s00702-021-02306-2 33624170 PMC7901515

[B34] De Caro C. Cristiano C. Avagliano C. Cuozzo M. La Rana G. Aviello G. (2020). Analgesic and anti-inflammatory effects of perampanel in acute and chronic pain models in mice: Interaction with the cannabinergic system. Front. Pharmacol. 11, 620221. 10.3389/fphar.2020.620221 33597883 PMC7883473

[B35] De Marchi E. Orioli E. Pegoraro A. Sangaletti S. Portararo P. Curti A. (2019). The P2X7 receptor modulates immune cells infiltration, ectonucleotidases expression and extracellular ATP levels in the tumor microenvironment. Oncogene 38 (19), 3636–3650. 10.1038/s41388-019-0684-y 30655604 PMC6756114

[B36] Dhaliwal J. S. Spurling B. C. Molla M. (2022). Duloxetine. Treasure Island (FL): StatPearls Publishing. 31747213

[B37] Diwo S. Petroianu G. (2002). “Die Pharmakologie von Ketamin: Enantiomere, Distomere, Eutomere und Razemat,” in S)-Ketamin: Aktuelle interdisziplinäre AspekteR. Klose & U. Hoppe. (Berlin, Heidelberg: Springer Berlin Heidelberg), 1–16.

[B38] Dogrul A. Ossipov M. H. Porreca F. (2009). Differential mediation of descending pain facilitation and inhibition by spinal 5HT-3 and 5HT-7 receptors. Brain Res. 1280, 52–59. 10.1016/j.brainres.2009.05.001 19427839

[B39] Dougherty J. A. Young H. Shafi T. (2002). Serotonin syndrome induced by amitriptyline, meperidine, and venlafaxine. Ann. Pharmacother. 36 (10), 1647–1648. 10.1345/aph.1C091 12243617

[B40] Drenth J. P. te Morsche R. H. Guillet G. Taieb A. Kirby R. L. Jansen J. B. (2005). SCN9A mutations define primary erythermalgia as a neuropathic disorder of voltage gated sodium channels. J. Invest. Dermatol 124 (6), 1333–1338. 10.1111/j.0022-202X.2005.23737.x 15955112

[B41] Duan L. Yang J. Slaughter M. M. (2009). Caffeine inhibition of ionotropic glycine receptors. J. Physiol. 587 (16), 4063–4075. 10.1113/jphysiol.2009.174797 19564396 PMC2756438

[B42] Duggan A. W. Hope P. J. Lang C. W. (1991). Microinjection of neuropeptide Y into the superficial dorsal horn reduces stimulus-evoked release of immunoreactive substance P in the anaesthetized cat. Neuroscience 44 (3), 733–740. 10.1016/0306-4522(91)90092-3 1721688

[B43] Dyer O. (2020). Purdue Pharma to plead guilty and pay $8.3bn over opioid marketing. Bmj 371, m4103. 10.1136/bmj.m4103 33093019

[B44] Edvinsson L. (2021). CGRP and migraine: From bench to bedside. Rev. Neurol. 177 (7), 785–790. 10.1016/j.neurol.2021.06.003 34275653

[B45] Egilman D. Collins G. Falender J. Shembo N. Keegan C. Tohan S. (2019). The marketing of OxyContin®: A cautionary tale. Indian J Med Ethics 4 (3), 183–193. 10.20529/ijme.2019.043 31727614

[B46] Eisenberg E. Pud D. (1998). Can patients with chronic neuropathic pain be cured by acute administration of the NMDA receptor antagonist amantadine? Pain 74 (2-3), 337–339. 10.1016/s0304-3959(97)00198-x 9520249

[B47] Emery E. C. Luiz A. P. Wood J. N. (2016). Nav1.7 and other voltage-gated sodium channels as drug targets for pain relief. Expert Opin. Ther. Targets 20 (8), 975–983. 10.1517/14728222.2016.1162295 26941184 PMC4950419

[B48] Fernandes E. S. Fernandes M. A. Keeble J. E. (2012). The functions of TRPA1 and TRPV1: Moving away from sensory nerves. Br. J. Pharmacol. 166 (2), 510–521. 10.1111/j.1476-5381.2012.01851.x 22233379 PMC3417484

[B49] Fertleman C. R. Baker M. D. Parker K. A. Moffatt S. Elmslie F. V. Abrahamsen B. (2006). SCN9A mutations in paroxysmal extreme pain disorder: Allelic variants underlie distinct channel defects and phenotypes. Neuron 52 (5), 767–774. 10.1016/j.neuron.2006.10.006 17145499

[B50] Finnerup N. B. Attal N. Haroutounian S. McNicol E. Baron R. Dworkin R. H. (2015). Pharmacotherapy for neuropathic pain in adults: A systematic review and meta-analysis. Lancet Neurol. 14 (2), 162–173. 10.1016/s1474-4422(14)70251-0 25575710 PMC4493167

[B51] Finnerup N. B. Otto M. McQuay H. J. Jensen T. S. Sindrup S. H. (2005). Algorithm for neuropathic pain treatment: An evidence based proposal. Pain 118 (3), 289–305. 10.1016/j.pain.2005.08.013 16213659

[B52] Fischer J. H. Barr A. N. Rogers S. L. Fischer P. A. Trudeau V. L. (1994). Lack of serious toxicity following gabapentin overdose. Neurology 44 (5), 982–983. 10.1212/wnl.44.5.982 8190316

[B53] Frediani B. Giusti A. Bianchi G. Dalle Carbonare L. Malavolta N. Cantarini L. (2018). Clodronate in the management of different musculoskeletal conditions. Minerva Med. 109 (4), 300–325. 10.23736/s0026-4806.18.05688-4 29947493

[B54] Fride E. Mechoulam R. (1993). Pharmacological activity of the cannabinoid receptor agonist, anandamide, a brain constituent. Eur. J. Pharmacol. 231 (2), 313–314. 10.1016/0014-2999(93)90468-W 8384116

[B55] Friedman R. A. Leon A. C. (2007). Expanding the black box - depression, antidepressants, and the risk of suicide. N. Engl. J. Med. 356 (23), 2343–2346. 10.1056/NEJMp078015 17485726

[B56] Froestl W. Muhs A. Pfeifer A. (2012). Cognitive enhancers (nootropics). Part 1: Drugs interacting with receptors. J. Alzheimers Dis. 32 (4), 793–887. 10.3233/jad-2012-121186 22886028

[B57] Fudin J. Boglish P. (2016). Ask The Expert: Is Tapentadol a Glorified Tramadol? Pract. Pain Manag 16 (1).

[B58] Gagnon M. Bergeron M. J. Lavertu G. Castonguay A. Tripathy S. Bonin R. P. (2013). Chloride extrusion enhancers as novel therapeutics for neurological diseases. Nat. Med. 19 (11), 1524–1528. 10.1038/nm.3356 24097188 PMC4005788

[B59] Gan S. H. Ismail R. Wan Adnan W. A. Zulmi W. (2007). Impact of CYP2D6 genetic polymorphism on tramadol pharmacokinetics and pharmacodynamics. Mol. Diagn Ther. 11 (3), 171–181. 10.1007/bf03256239 17570739

[B60] Girard P. Chauvin M. Verleye M. (2016). Nefopam analgesia and its role in multimodal analgesia: A review of preclinical and clinical studies. Clin. Exp. Pharmacol. Physiol. 43 (1), 3–12. 10.1111/1440-1681.12506 26475417

[B61] Goadsby P. J. Edvinsson L. (1993). The trigeminovascular system and migraine: Studies characterizing cerebrovascular and neuropeptide changes seen in humans and cats. Ann. Neurol. 33 (1), 48–56. 10.1002/ana.410330109 8388188

[B62] Goldberg Y. P. MacFarlane J. MacDonald M. L. Thompson J. Dube M. P. Mattice M. (2007). Loss-of-function mutations in the Nav1.7 gene underlie congenital indifference to pain in multiple human populations. Clin. Genet. 71 (4), 311–319. 10.1111/j.1399-0004.2007.00790.x 17470132

[B63] Gouin O. L'Herondelle K. Lebonvallet N. Le Gall-Ianotto C. Sakka M. Buhé V. (2017). TRPV1 and TRPA1 in cutaneous neurogenic and chronic inflammation: Pro-inflammatory response induced by their activation and their sensitization. Protein Cell 8 (9), 644–661. 10.1007/s13238-017-0395-5 28364279 PMC5563280

[B64] Greenberg D. A. (2003). “Endocannabinoids,” in Encyclopedia of the neurological sciences. Editors Aminoff M. J. Daroff R. B. (New York: Academic Press), 143–144.

[B65] Grzegorzewski J. Bartsch F. Köller A. König M. (2021). Pharmacokinetics of caffeine: A systematic analysis of reported data for application in metabolic phenotyping and liver function testing. Front. Pharmacol. 12, 752826. 10.3389/fphar.2021.752826 35280254 PMC8914174

[B66] Habib A. M. Okorokov A. L. Hill M. N. Bras J. T. Lee M.-C. Li S. (2019). Microdeletion in a FAAH pseudogene identified in a patient with high anandamide concentrations and pain insensitivity. Br. J. Anaesth. 123 (2), e249–e253. 10.1016/j.bja.2019.02.019 30929760 PMC6676009

[B67] Hasuzawa N. Moriyama S. Moriyama Y. Nomura M. (2020). Physiopathological roles of vesicular nucleotide transporter (VNUT), an essential component for vesicular ATP release. Biochim. Biophys. Acta Biomembr. 1862 (12), 183408. 10.1016/j.bbamem.2020.183408 32652056

[B68] Heel R. C. Brogden R. N. Pakes G. E. Speight T. M. Avery G. S. (1980). Nefopam: A review of its pharmacological properties and therapeutic efficacy. Drugs 19 (4), 249–267. 10.2165/00003495-198019040-00001 6991238

[B69] Hensgens M. P. Goorhuis A. Dekkers O. M. Kuijper E. J. (2012). Time interval of increased risk for *Clostridium difficile* infection after exposure to antibiotics. J. Antimicrob. Chemother. 67 (3), 742–748. 10.1093/jac/dkr508 22146873

[B70] Inoue K. (2017). Purinergic signaling in microglia in the pathogenesis of neuropathic pain. Proc. Jpn. Acad. Ser. B Phys. Biol. Sci. 93 (4), 174–182. 10.2183/pjab.93.011 PMC5489427 28413195

[B71] Jensen T. S. Høye K. Fricová J. Vanelderen P. Ernault E. Siciliano T. (2014). Tolerability of the capsaicin 8% patch following pretreatment with lidocaine or tramadol in patients with peripheral neuropathic pain: A multicentre, randomized, assessor-blinded study. Eur. J. Pain 18 (9), 1240–1247. 10.1002/j.1532-2149.2014.00479.x 24664539 PMC4232045

[B72] Jeon Y. T. Oh A. Y. Jin S. J. Park B. S. Choi E. S. (2019). Effects of intraoperative nefopam on catheter-related bladder discomfort in patients undergoing robotic nephrectomy: A randomized double-blind study. J. Clin. Med. 8 (4), 519. 10.3390/jcm8040519 30995766 PMC6518107

[B73] Jung S. M. Peyton L. Essa H. Choi D. S. (2022). Adenosine receptors: Emerging non-opioids targets for pain medications. Neurobiol. Pain 11, 100087. 10.1016/j.ynpai.2022.100087 35372716 PMC8971635

[B74] Kahle K. T. Khanna A. Clapham D. E. Woolf C. J. (2014). Therapeutic restoration of spinal inhibition via druggable enhancement of potassium-chloride cotransporter KCC2-mediated chloride extrusion in peripheral neuropathic pain. JAMA Neurol. 71 (5), 640–645. 10.1001/jamaneurol.2014.21 24615367 PMC4465580

[B75] Kanellopoulos J. M. Almeida-da-Silva C. L. C. Rüütel Boudinot S. Ojcius D. M. (2021). Structural and functional features of the P2X4 receptor: An immunological perspective. Front. Immunol. 12, 645834. 10.3389/fimmu.2021.645834 33897694 PMC8059410

[B76] Kappelmann N. Lewis G. Dantzer R. Jones P. B. Khandaker G. M. (2018). Antidepressant activity of anti-cytokine treatment: A systematic review and meta-analysis of clinical trials of chronic inflammatory conditions. Mol. Psychiatry 23 (2), 335–343. 10.1038/mp.2016.167 27752078 PMC5794896

[B77] Kato Y. Hiasa M. Ichikawa R. Hasuzawa N. Kadowaki A. Iwatsuki K. (2017). Identification of a vesicular ATP release inhibitor for the treatment of neuropathic and inflammatory pain. Proc. Natl. Acad. Sci. U. S. A. 114 (31), E6297–e6305. 10.1073/pnas.1704847114 28720702 PMC5547629

[B78] Kaye A. D. Chernobylsky D. J. Thakur P. Siddaiah H. Kaye R. J. Eng L. K. (2020). Dexmedetomidine in enhanced recovery after surgery (ERAS) protocols for postoperative pain. Curr. Pain Headache Rep. 24 (5), 21. 10.1007/s11916-020-00853-z 32240402 PMC7223065

[B79] Keating G. M. (2015). Diquafosol ophthalmic solution 3 %: a review of its use in dry eye. Drugs 75 (8), 911–922. 10.1007/s40265-015-0409-7 25968930

[B80] Kim K. H. Abdi S. (2014). Rediscovery of nefopam for the treatment of neuropathic pain. Korean J. Pain 27 (2), 103–111. 10.3344/kjp.2014.27.2.103 24748937 PMC3990817

[B81] Kim S. K. Min B.-I. Kim J. H. Hwang B. G. Yoo G. Y. Park D. S. (2005). Effects of alpha1-and alpha2-adrenoreceptor antagonists on cold allodynia in a rat tail model of neuropathic pain. Brain Res. 1039 (1), 207–210. 10.1016/j.brainres.2005.01.051 15781064

[B82] Kohno K. Tsuda M. (2021). Role of microglia and P2X4 receptors in chronic pain. Pain Rep. 6 (1), e864. 10.1097/pr9.0000000000000864 33981920 PMC8108579

[B83] Kornhuber J. Kennepohl E. M. Bleich S. Wiltfang J. Kraus T. Reulbach U. (2007). Memantine pharmacotherapy: A naturalistic study using a population pharmacokinetic approach. Clin. Pharmacokinet. 46 (7), 599–612. 10.2165/00003088-200746070-00005 17596105

[B84] Kornhuber J. Mack-Burkhardt F. Riederer P. Hebenstreit G. F. Reynolds G. P. Andrews H. B. (1989). [3H]MK-801 binding sites in postmortem brain regions of schizophrenic patients. J. Neural Transm. 77 (2-3), 231–236. 10.1007/bf01248936 2547892

[B85] Lanzetti S. Di Biase V. (2022). Small molecules as modulators of voltage-gated calcium channels in neurological disorders: State of the Art and perspectives. Molecules 27 (4), 1312. 10.3390/molecules27041312 35209100 PMC8879281

[B86] Lee G. Grovey B. Furnish T. Wallace M. (2018). Medical cannabis for neuropathic pain. Curr. Pain Headache Rep. 22 (1), 8. 10.1007/s11916-018-0658-8 29388063

[B87] Lee H. G. Kim W. M. Kim J. M. Bae H. B. Choi J. I. (2015). Intrathecal nefopam-induced antinociception through activation of descending serotonergic projections involving spinal 5-HT7 but not 5-HT3 receptors. Neurosci. Lett. 587, 120–125. 10.1016/j.neulet.2014.12.040 25534502

[B88] Leon A. C. (2007). The revised warning for antidepressants and suicidality: Unveiling the black box of statistical analyses. Am. J. Psychiatry 164 (12), 1786–1789. 10.1176/appi.ajp.2007.07050775 18056231

[B89] Lin J. P. Chen C. Q. Huang L. E. Li N. N. Yang Y. Zhu S. M. (2018). Dexmedetomidine attenuates neuropathic pain by inhibiting P2X7R expression and ERK phosphorylation in rats. Exp. Neurobiol. 27 (4), 267–276. 10.5607/en.2018.27.4.267 30181689 PMC6120967

[B90] Liu Q. Q. Yao X. X. Gao S. H. Li R. Li B. J. Yang W. (2020). Role of 5-HT receptors in neuropathic pain: Potential therapeutic implications. Pharmacol. Res. 159, 104949. 10.1016/j.phrs.2020.104949 32464329

[B91] Liu X. Ma L. Zhang S. Ren Y. Dirksen R. T. (2017). CD73 controls extracellular adenosine generation in the trigeminal nociceptive nerves. J. Dent. Res. 96 (6), 671–677. 10.1177/0022034517692953 28530470 PMC5444617

[B92] Magni G. Merli D. Verderio C. Abbracchio M. P. Ceruti S. (2015). P2Y2 receptor antagonists as anti-allodynic agents in acute and sub-chronic trigeminal sensitization: Role of satellite glial cells. Glia 63 (7), 1256–1269. 10.1002/glia.22819 25779655

[B93] Meng H. Dai T. Hanlon J. G. Downar J. Alibhai S. M. H. Clarke H. (2020). Cannabis and cannabinoids in cancer pain management. Curr. Opin. Support Palliat. Care 14 (2), 87–93. 10.1097/spc.0000000000000493 32332209

[B94] Mihara S. Shibamoto T. (2015). The role of flavor and fragrance chemicals in TRPA1 (transient receptor potential cation channel, member A1) activity associated with allergies. Allergy Asthma Clin. Immunol. 11 (1), 11. 10.1186/s13223-015-0074-0 25897313 PMC4404258

[B95] Minett M. S. Nassar M. A. Clark A. K. Passmore G. Dickenson A. H. Wang F. (2012). Distinct Nav1.7-dependent pain sensations require different sets of sensory and sympathetic neurons. Nat. Commun. 3, 791. 10.1038/ncomms1795 22531176 PMC3337979

[B96] Miras-Portugal M. T. Menéndez-Méndez A. Gómez-Villafuertes R. Ortega F. Delicado E. G. Pérez-Sen R. (2019). Physiopathological role of the vesicular nucleotide transporter (VNUT) in the central nervous system: Relevance of the vesicular nucleotide release as a potential therapeutic target. Front. Cell Neurosci. 13, 224. 10.3389/fncel.2019.00224 31156398 PMC6533569

[B97] Mishra A. Behura A. Kumar A. Naik L. Swain A. Das M. (2021). P2X7 receptor in multifaceted cellular signalling and its relevance as a potential therapeutic target in different diseases. Eur. J. Pharmacol. 906, 174235. 10.1016/j.ejphar.2021.174235 34097884

[B98] Miyahara Y. Funahashi H. Naono-Nakayama R. Haruta-Tsukamoto A. Nishimori T. Ishida Y. (2019). Role of serotonin and noradrenaline in the acute itch processing in mice. Eur. J. Pharmacol. 850, 118–125. 10.1016/j.ejphar.2019.02.013 30763572

[B99] Mlost J. Bryk M. Starowicz K. (2020). Cannabidiol for pain treatment: Focus on pharmacology and mechanism of action. Int. J. Mol. Sci. 21 (22), 8870. 10.3390/ijms21228870 33238607 PMC7700528

[B100] Montgomery S. A. Baldwin D. Green M. (1989). Why do amitriptyline and dothiepin appear to be so dangerous in overdose? Acta Psychiatr. Scand. Suppl. 354, 47–53. 10.1111/j.1600-0447.1989.tb03046.x 2589103

[B101] Moore R. A. Chi C. C. Wiffen P. J. Derry S. Rice A. S. (2015). Oral nonsteroidal anti-inflammatory drugs for neuropathic pain. Cochrane Database Syst. Rev. 2015 (10), Cd010902. 10.1002/14651858.CD010902.pub2 26436601 PMC6481590

[B102] Morel V. Pickering M. E. Goubayon J. Djobo M. Macian N. Pickering G. (2021). Magnesium for pain treatment in 2021? State of the Art. Nutrients 13 (5), 1397. 10.3390/nu13051397 33919346 PMC8143286

[B103] Mücke M. Phillips T. Radbruch L. Petzke F. Häuser W. (2018). Cannabis-based medicines for chronic neuropathic pain in adults. Cochrane Database Syst. Rev. 3 (3), Cd012182. 10.1002/14651858.CD012182.pub2 29513392 PMC6494210

[B104] Murakami T. Fujihara T. Horibe Y. Nakamura M. (2004). Diquafosol elicits increases in net Cl-transport through P2Y2 receptor stimulation in rabbit conjunctiva. Ophthalmic Res. 36 (2), 89–93. 10.1159/000076887 15017104

[B105] Murgia M. Hanau S. Pizzo P. Rippa M. Di Virgilio F. (1993). Oxidized ATP. An irreversible inhibitor of the macrophage purinergic P2Z receptor. J. Biol. Chem. 268 (11), 8199–8203. 10.1016/s0021-9258(18)53082-9 8463330

[B106] Na H. S. Ryu J. H. Do S. H. (2011). “The role of magnesium in pain,” in Magnesium in the central nervous system. Editors Vink R. Nechifor M. (Adelaide (AU): University of Adelaide Press). 29920000

[B107] Nair A. S. Sahoo R. K. (2019). Efficacy of memantine hydrochloride in neuropathic pain. Indian J. Palliat. Care 25 (1), 161–162. 10.4103/ijpc.ijpc_189_18 30820121 PMC6388583

[B108] Nam K. Kim H. J. Yoo A. (2019). Efficacy and safety of topical 3% diquafosol ophthalmic solution for the treatment of multifactorial dry eye disease: Meta-analysis of randomized clinical trials. Ophthalmic Res. 61 (4), 188–198. 10.1159/000492896 30654362

[B109] Nelson T. S. Taylor B. K. (2021). Targeting spinal neuropeptide Y1 receptor-expressing interneurons to alleviate chronic pain and itch. Prog. Neurobiol. 196, 101894. 10.1016/j.pneurobio.2020.101894 32777329 PMC8088728

[B110] Niesters M. Martini C. Dahan A. (2014). Ketamine for chronic pain: Risks and benefits. Br. J. Clin. Pharmacol. 77 (2), 357–367. 10.1111/bcp.12094 23432384 PMC4014022

[B111] Niimi A. Saito J. Kamei T. Shinkai M. Ishihara H. Machida M. (2022). Randomised trial of the P2X(3) receptor antagonist sivopixant for refractory chronic cough. Eur. Respir. J. 59 (6), 2100725. 10.1183/13993003.00725-2021 34649978 PMC9176336

[B112] Noe C. E. (2020). Pain management for clinicians A guide to assessment and treatment. Switzerland: Springer Nature.

[B113] Nörenberg W. Sobottka H. Hempel C. Plötz T. Fischer W. Schmalzing G. (2012). Positive allosteric modulation by ivermectin of human but not murine P2X7 receptors. Br. J. Pharmacol. 167 (1), 48–66. 10.1111/j.1476-5381.2012.01987.x 22506590 PMC3448913

[B114] O'Brien J. J. O'Callaghan J. P. Miller D. B. Chalgeri S. Wennogle L. P. Davis R. E. (2020). Inhibition of calcium-calmodulin-dependent phosphodiesterase (PDE1) suppresses inflammatory responses. Mol. Cell Neurosci. 102, 103449. 10.1016/j.mcn.2019.103449 31770590 PMC7783477

[B115] Obad A. Peeran A. Little J. I. Haddad G. E. Tarzami S. T. (2018). Alcohol-mediated organ damages: Heart and brain. Front. Pharmacol. 9, 81. 10.3389/fphar.2018.00081 29487525 PMC5816804

[B116] Onda A. Murata Y. Rydevik B. Larsson K. Kikuchi S. Olmarker K. (2005). Nerve growth factor content in dorsal root ganglion as related to changes in pain behavior in a rat model of experimental lumbar disc herniation. Spine (Phila Pa 1976) 30 (2), 188–193. 10.1097/01.brs.0000150830.12518.26 15644754

[B117] Orita S. Ishikawa T. Miyagi M. Ochiai N. Inoue G. Eguchi Y. (2011). Pain-related sensory innervation in monoiodoacetate-induced osteoarthritis in rat knees that gradually develops neuronal injury in addition to inflammatory pain. BMC Musculoskelet. Disord. 12, 134. 10.1186/1471-2474-12-134 21679434 PMC3142251

[B118] Orrego M. Jiménez-Rodriguez A. Osorio-Forero A. Restrepo F. Arias J. Tamayo L. (2016). Different brain structures exhibit the same caffeine levels after the administration of A single dose of caffeine. Biosalud 15, 20–27. 10.17151/biosa.2016.15.2.3

[B119] Ortíz-Rentería M. Juárez-Contreras R. González-Ramírez R. Islas L. D. Sierra-Ramírez F. Llorente I. (2018). TRPV1 channels and the progesterone receptor Sig-1R interact to regulate pain. Proc. Natl. Acad. Sci. U. S. A. 115 (7), E1657–e1666. 10.1073/pnas.1715972115 29378958 PMC5816171

[B120] Park Y. S. Kim Y. B. Kim J. M. (2014). Status epilepticus caused by nefopam. J. Korean Neurosurg. Soc. 56 (5), 448–450. 10.3340/jkns.2014.56.5.448 25535527 PMC4273008

[B121] Pastor-Anglada M. Pérez-Torras S. (2018). Who is who in adenosine transport. Front. Pharmacol. 9, 627. 10.3389/fphar.2018.00627 29962948 PMC6010718

[B122] Patel R. Dickenson A. H. (2022). Neuropharmacological basis for multimodal analgesia in chronic pain. Postgrad. Med. 134 (3), 245–259. 10.1080/00325481.2021.1985351 34636261

[B123] Perez-Lloret S. Rascol O. (2018). Efficacy and safety of amantadine for the treatment of L-DOPA-induced dyskinesia. J. Neural Transm. (Vienna) 125 (8), 1237–1250. 10.1007/s00702-018-1869-1 29511826

[B124] Petersen K. U. (2013). Caffeine in analgesics--myth or medicine? MMW Fortschr Med. 155 (4), 109–114. 10.1007/s15006-013-2541-1 24934064

[B125] Peterson A. R. Binder D. K. (2019). Post-translational regulation of GLT-1 in neurological diseases and its potential as an effective therapeutic target. Front. Mol. Neurosci. 12, 164. 10.3389/fnmol.2019.00164 31338020 PMC6629900

[B126] Piper S. N. Röhm K. D. Suttner S. W. Maleck W. H. Kranke P. Boldt J. (2004). A comparison of nefopam and clonidine for the prevention of postanaesthetic shivering: A comparative, double-blind and placebo-controlled dose-ranging study. Anaesthesia 59 (6), 559–564. 10.1111/j.1365-2044.2004.03734.x 15144295

[B127] Piper S. N. Schmidt C. C. Suttner S. W. Kumle B. Triem J. G. Maleck W. H. (1998). Prophylactic nefopam administration for post-anesthetic shivering. Anasthesiol Intensivmed. Notfallmed Schmerzther 33 (12), 786–789. 10.1055/s-2007-994854 9893913

[B128] Romero-Sandoval E. A. Fincham J. E. Kolano A. L. Sharpe B. N. Alvarado-Vázquez P. A. (2018). Cannabis for chronic pain: Challenges and considerations. Pharmacotherapy 38 (6), 651–662. 10.1002/phar.2115 29637590

[B129] Romero-Sandoval E. A. Kolano A. L. Alvarado-Vázquez P. A. (2017). Cannabis and cannabinoids for chronic pain. Curr. Rheumatol. Rep. 19 (11), 67. 10.1007/s11926-017-0693-1 28983880

[B130] Rotondo J. C. Mazziotta C. Lanzillotti C. Stefani C. Badiale G. Campione G. (2022). The role of purinergic P2X7 receptor in inflammation and cancer: Novel molecular insights and clinical applications. Cancers (Basel) 14 (5), 1116. 10.3390/cancers14051116 35267424 PMC8909580

[B131] Roulet L. Rollason V. Desmeules J. Piguet V. (2021). Tapentadol versus tramadol: A narrative and comparative review of their pharmacological, efficacy and safety profiles in adult patients. Drugs 81 (11), 1257–1272. 10.1007/s40265-021-01515-z 34196947 PMC8318929

[B132] Russell F. A. King R. Smillie S.-J. Kodji X. Brain S. D. (2014). Calcitonin gene-related peptide: Physiology and pathophysiology. Physiol. Rev. 94 (4), 1099–1142. 10.1152/physrev.00034.2013 25287861 PMC4187032

[B133] Salas M. M. Clifford J. L. Hayden J. R. Iadarola M. J. Averitt D. L. (2017). Local resiniferatoxin induces long-lasting analgesia in a rat model of full thickness thermal injury. Pain Med. 18 (12), 2453–2465. 10.1093/pm/pnw260 27794548 PMC6279302

[B134] Saleem H. Tovey S. C. Molinski T. F. Taylor C. W. (2014). Interactions of antagonists with subtypes of inositol 1, 4, 5-trisphosphate (IP3) receptor. Br. J. Pharmacol. 171 (13), 3298–3312. 10.1111/bph.12685 24628114 PMC4080982

[B135] Sanacora G. Schatzberg A. F. (2015). Ketamine: Promising path or false prophecy in the development of novel therapeutics for mood disorders? Neuropsychopharmacology 40 (2), 259–267. 10.1038/npp.2014.261 25257213 PMC4443967

[B136] Savio L. E. B. de Andrade Mello P. da Silva C. G. Coutinho-Silva R. (2018). The P2X7 receptor in inflammatory diseases: Angel or demon? Front. Pharmacol. 9, 52. 10.3389/fphar.2018.00052 29467654 PMC5808178

[B137] Sawynok J. (2016). Adenosine receptor targets for pain. Neuroscience 338, 1–18. 10.1016/j.neuroscience.2015.10.031 26500181

[B138] Schwedt T. J. Garza M. (2022). Preventive treatment of episodic migraine in adults. Available at: https://www-uptodate-com.libconnect.ku.ac.ae/contents/preventive-treatment-of-episodic-migraine-in-adults?search=erenumab&source=search_result&selectedTitle=2∼5&usage_type=default&display_rank=1#H2010410496 (Accessed 05 12, 2022).

[B139] Sheets P. L. Jackson J. O. 2nd Waxman S. G. Dib-Hajj S. D. Cummins T. R. (2007). A Nav1.7 channel mutation associated with hereditary erythromelalgia contributes to neuronal hyperexcitability and displays reduced lidocaine sensitivity. J. Physiol. 581 (3), 1019–1031. 10.1113/jphysiol.2006.127027 17430993 PMC2170829

[B140] Shi C. Das V. Li X. Kc R. Qiu S. I O. S. (2018). Development of an *in vivo* mouse model of discogenic low back pain. J. Cell Physiol. 233 (10), 6589–6602. 10.1002/jcp.26280 29150945

[B141] Shin H. J. Na H. S. Do S. H. (2020). Magnesium and pain. Nutrients 12 (8), 2184. 10.3390/nu12082184 32718032 PMC7468697

[B142] Singh D. Nag K. Shetti A. Krishnaveni N. (2013). Tapentadol hydrochloride: A novel analgesic. Saudi J. Anaesth. 7 (3), 322–326. 10.4103/1658-354x.115319 24015138 PMC3757808

[B143] Sisignano M. Gribbon P. Geisslinger G. (2022). Drug repurposing to target neuroinflammation and sensory neuron-dependent pain. Drugs 82 (4), 357–373. 10.1007/s40265-022-01689-0 35254645 PMC8899787

[B144] Smith H. S. (2012). Opioids and neuropathic pain. Pain Physician 15, Es93–110. 10.36076/ppj.2012/15/es93 22786465

[B145] Smith T. Nicholson R. A. (2007). Review of duloxetine in the management of diabetic peripheral neuropathic pain. Vasc. Health Risk Manag. 3 (6), 833–844. 18200804 PMC2350145

[B146] Soós R. Gyebrovszki Á. Tóth Á. Jeges S. Wilhelm M. (2021). Effects of caffeine and caffeinated beverages in children, adolescents and young adults: Short review. Int. J. Environ. Res. Public Health 18 (23), 12389. 10.3390/ijerph182312389 34886115 PMC8656548

[B147] Sowa N. A. Taylor-Blake B. Zylka M. J. (2010). Ecto-5'-nucleotidase (CD73) inhibits nociception by hydrolyzing AMP to adenosine in nociceptive circuits. J. Neurosci. 30 (6), 2235–2244. 10.1523/jneurosci.5324-09.2010 20147550 PMC2826808

[B148] Staruschenko A. Jeske N. A. Akopian A. N. (2010). Contribution of TRPV1-TRPA1 interaction to the single channel properties of the TRPA1 channel. J. Biol. Chem. 285 (20), 15167–15177. 10.1074/jbc.M110.106153 20231274 PMC2865321

[B149] Stokes L. Layhadi J. A. Bibic L. Dhuna K. Fountain S. J. (2017). P2X4 receptor function in the nervous system and current breakthroughs in pharmacology. Front. Pharmacol. 8, 291. 10.3389/fphar.2017.00291 28588493 PMC5441391

[B150] Story G. M. Peier A. M. Reeve A. J. Eid S. R. Mosbacher J. Hricik T. R. (2003). ANKTM1, a TRP-like channel expressed in nociceptive neurons, is activated by cold temperatures. Cell 112 (6), 819–829. 10.1016/s0092-8674(03)00158-2 12654248

[B151] Subedi M. Bajaj S. Kumar M. S. Yc M. (2019). An overview of tramadol and its usage in pain management and future perspective. Biomed. Pharmacother. 111, 443–451. 10.1016/j.biopha.2018.12.085 30594783

[B152] Suri S. Mönkkönen J. Taskinen M. Pesonen J. Blank M. A. Phipps R. J. (2001). Nitrogen-containing bisphosphonates induce apoptosis of caco-2 cells *in vitro* by inhibiting the mevalonate pathway: A model of bisphosphonate-induced gastrointestinal toxicity. Bone 29 (4), 336–343. 10.1016/S8756-3282(01)00589-0 11595616

[B153] Tozaki-Saitoh H. Takeda H. Inoue K. (2022). The role of microglial purinergic receptors in pain signaling. Molecules 27 (6), 1919. 10.3390/molecules27061919 35335282 PMC8949888

[B154] Upadhya M. A. Dandekar M. P. Kokare D. M. Singru P. S. Subhedar N. K. (2009). Involvement of neuropeptide Y in the acute, chronic and withdrawal responses of morphine in nociception in neuropathic rats: Behavioral and neuroanatomical correlates. Neuropeptides 43 (4), 303–314. 10.1016/j.npep.2009.05.003 19556004

[B155] Urits I. Gress K. Charipova K. Habib K. Lee D. Lee C. (2020). Use of cannabidiol (CBD) for the treatment of chronic pain. Best. Pract. Res. Clin. Anaesthesiol. 34 (3), 463–477. 10.1016/j.bpa.2020.06.004 33004159

[B156] Vane J. R. Botting R. M. (1998). Anti-inflammatory drugs and their mechanism of action. Inflamm. Res. 47 (2), S78–S87. 10.1007/s000110050284 9831328

[B157] Vazzana M. Andreani T. Fangueiro J. Faggio C. Silva C. Santini A. (2015). Tramadol hydrochloride: Pharmacokinetics, pharmacodynamics, adverse side effects, co-administration of drugs and new drug delivery systems. Biomed. Pharmacother. 70, 234–238. 10.1016/j.biopha.2015.01.022 25776506

[B158] Vincenzi F. Pasquini S. Borea P. A. Varani K. (2020). Targeting adenosine receptors: A potential pharmacological avenue for acute and chronic pain. Int. J. Mol. Sci. 21 (22), 8710. 10.3390/ijms21228710 33218074 PMC7698931

[B159] White J. R. Jr. Padowski J. M. Zhong Y. Chen G. Luo S. Lazarus P. (2016). Pharmacokinetic analysis and comparison of caffeine administered rapidly or slowly in coffee chilled or hot versus chilled energy drink in healthy young adults. Clin. Toxicol. (Phila) 54 (4), 308–312. 10.3109/15563650.2016.1146740 27100333 PMC4898153

[B160] Wiesenfeld-Hallin Z. Xu X. J. Hökfelt T. (2002). The role of spinal cholecystokinin in chronic pain states. Pharmacol. Toxicol. 91 (6), 398–403. 10.1034/j.1600-0773.2002.910619.x 12688385

[B161] Wikipedia (2022). Glutamate transporter. Available at: https://en.wikipedia.org/wiki/Glutamate_transporter (Accessed October 1, 2022).

[B162] Wikoff D. Welsh B. T. Henderson R. Brorby G. P. Britt J. Myers E. (2017). Systematic review of the potential adverse effects of caffeine consumption in healthy adults, pregnant women, adolescents, and children. Food Chem. Toxicol. 109, 585–648. 10.1016/j.fct.2017.04.002 28438661

[B163] Yamashita T. Yamamoto S. Zhang J. Kometani M. Tomiyama D. Kohno K. (2016). Duloxetine inhibits microglial P2X4 receptor function and alleviates neuropathic pain after peripheral nerve injury. PLoS One 11 (10), e0165189. 10.1371/journal.pone.0165189 27768754 PMC5074465

[B164] Yancey J. R. Dattoli G. (2013). Caffeine as an analgesic adjuvant for acute pain in adults. Am. Fam. Physician 87 (1), 11. 23317021

[B165] Yang Y. Li Q. He Q. H. Han J. S. Su L. Wan Y. (2018). Heteromerization of μ-opioid receptor and cholecystokinin B receptor through the third transmembrane domain of the μ-opioid receptor contributes to the anti-opioid effects of cholecystokinin octapeptide. Exp. Mol. Med. 50 (5), 1–16. 10.1038/s12276-018-0090-5 PMC5960647 29780163

[B166] Yang Y. Wang Y. Li S. Xu Z. Li H. Ma L. (2004). Mutations in SCN9A, encoding a sodium channel alpha subunit, in patients with primary erythermalgia. J. Med. Genet. 41 (3), 171–174. 10.1136/jmg.2003.012153 14985375 PMC1735695

[B167] Yeo M. Chen Y. Jiang C. Chen G. Wang K. Chandra S. (2021). Repurposing cancer drugs identifies kenpaullone which ameliorates pathologic pain in preclinical models via normalization of inhibitory neurotransmission. Nat. Commun. 12 (1), 6208. 10.1038/s41467-021-26270-3 34707084 PMC8551327

[B168] Yoshimura M. Furue H. (2006). Mechanisms for the anti-nociceptive actions of the descending noradrenergic and serotonergic systems in the spinal cord. J. Pharmacol. Sci. 101 (2), 107–117. 10.1254/jphs.crj06008x 16766858

[B169] Zhang W. J. Luo H. L. Zhu Z. M. (2020). The role of P2X4 receptors in chronic pain: A potential pharmacological target. Biomed. Pharmacother. 129, 110447. 10.1016/j.biopha.2020.110447 32887026

[B170] Zhao Y. He J. Yu N. Jia C. Wang S. (2020). Mechanisms of dexmedetomidine in neuropathic pain. Front. Neurosci. 14, 330. 10.3389/fnins.2020.00330 32431587 PMC7214625

[B171] Zylka M. J. (2011). Pain-relieving prospects for adenosine receptors and ectonucleotidases. Trends Mol. Med. 17 (4), 188–196. 10.1016/j.molmed.2010.12.006 21236731 PMC3078941

